# Development of deaminase-free T-to-S base editor and C-to-G base editor by engineered human uracil DNA glycosylase

**DOI:** 10.1038/s41467-024-49343-5

**Published:** 2024-06-08

**Authors:** Huawei Tong, Haoqiang Wang, Xuchen Wang, Nana Liu, Guoling Li, Danni Wu, Yun Li, Ming Jin, Hengbin Li, Yinghui Wei, Tong Li, Yuan Yuan, Linyu Shi, Xuan Yao, Yingsi Zhou, Hui Yang

**Affiliations:** 1 HuidaGene Therapeutics Co., Ltd., Shanghai, China; 2 Institute of Neuroscience, State Key Laboratory of Neuroscience, Key Laboratory of Primate Neurobiology, Center for Excellence in Brain Science and Intelligence Technology, Chinese Academy of Sciences, Shanghai, China; 3 College of Life Sciences, University of Chinese Academy of Sciences, Beijing, China; 4 Department of Neurology and Institute of Neurology of First Affiliated Hospital, Institute of Neuroscience, and Fujian Key Laboratory of Molecular Neurology, Fujian Medical University, Fuzhou, China; 5 International Joint Agriculture Research Center for Animal Bio-Breeding of Ministry of Agriculture and Rural Affairs, College of Animal Science and Technology, Northwest A&F University, Yangling, Shaanxi China; 6 School of Future Technology on Bio-Breeding, College of Animal Science and Technology, Northwest A&F University, Yangling, Shaanxi China

**Keywords:** CRISPR-Cas9 genome editing, Molecular engineering, Genetic engineering

## Abstract

DNA base editors enable direct editing of adenine (A), cytosine (C), or guanine (G), but there is no base editor for direct thymine (T) editing currently. Here we develop two deaminase-free glycosylase-based base editors for direct T editing (gTBE) and C editing (gCBE) by fusing Cas9 nickase (nCas9) with engineered human uracil DNA glycosylase (UNG) variants. By several rounds of structure-informed rational mutagenesis on UNG in cultured human cells, we obtain gTBE and gCBE with high activity of T-to-S (i.e., T-to-C or T-to-G) and C-to-G conversions, respectively. Furthermore, we conduct parallel comparison of gTBE/gCBE with those recently developed using other protein engineering strategies, and find gTBE/gCBE show the outperformance. Thus, we provide several base editors, gTBEs and gCBEs, with corresponding engineered UNG variants, broadening the targeting scope of base editors.

## Abstract (web summary)

Efficient base editors for direct thymine (T) editing are highly desirable. Here, authors develop two deaminase-free glycosylase-based base editors for direct T editing (gTBE) and C editing (gCBE) by rounds of structure-informed mutagenesis on human DNA glycosylase UNG and further engineering.

## Introduction

Base editors enable single-nucleotide edits with high precision and efficiency, providing powerful tools for the fields of life science and medicine^1,2^. Two categories of DNA base editors, deaminase-based base editor (dBE) and deaminase-free glycosylase-based base editor (gBE), have been developed to date^3^. The dBEs perform base editing using single-stranded DNA (ssDNA) or double-stranded DNA (dsDNA) deaminase enzymes, such as the evolved tRNA adenosine deaminase TadA, AID/APOBEC-like cytidine deaminase and double-stranded DNA deaminase toxin A (DddA) variants. The deamination of A or C as an essential step is required for all dBEs, including adenine base editor (ABE)^4^, cytosine base editor (CBE)^5^, DddA-derived cytosine base editor (DdCBE)^6,7^, and their derivatives (e.g., A&C-BEmax^8^, AYBE^9^, AXBE/ACBE^10^ and CGBEs^11–15^). Recently, we have developed a gBE enabling direct G editing (i.e., deaminase-free glycosylase-based guanine base editor, gGBE)^3^, based on engineered human N-methylpurine DNA glycosylase (MPG; also known as alkyladenine DNA glycosylase, AAG). So far, dBEs and gGBE could enable editing of adenine (A), cytosine (C), or guanine (G), but no base editor for thymine (T) editing is available now. Base conversion by deamination is impossible for T (due to the absence of amine), making the development of thymine base editor still challenged.

Here, we develop a deaminase-free glycosylase-based thymine base editor (gTBE) as well as a deaminase-free glycosylase-based cytosine base editor (gCBE), to achieve orthogonal base editing, that is, gTBE for direct T editing and gCBE for direct C editing, respectively. After several rounds of mutagenesis of the uracil DNA glycosylase (UNG, or UDG) moieties, we obtain marked enhancement of editing activity for T editing and C editing, as compared with that obtained by wild-type (WT) UNG variant. We characterize the editing profile of gTBE and gCBE by targeting dozens of endogenous genomic loci in cultured mammalian cells as well as mouse embryos, demonstrating their high base editing efficiency.

## Results

### Development of orthogonal base editors based on engineered glycosylases

**Fig. 1:**
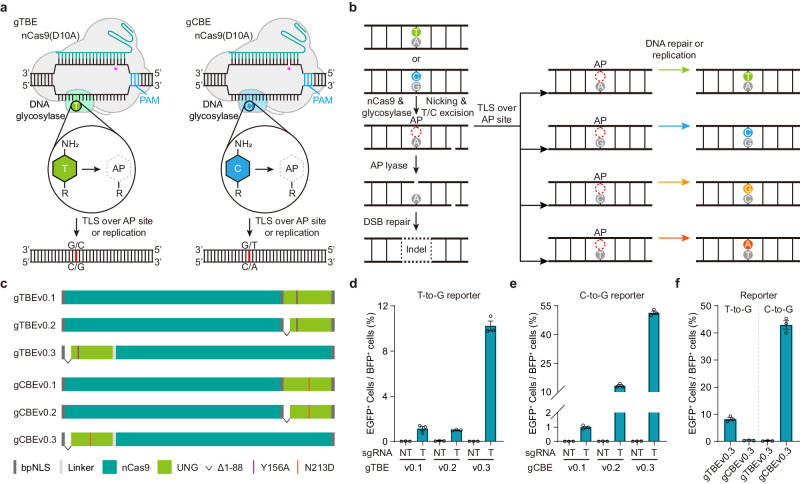
Design and mechanisms of two orthogonal glycosylase-based base editors. **a** Prototype versions of a deaminase-free glycosylase-based thymine base editor (gTBE) and a deaminase-free glycosylase-based cytosine base editor (gCBE). PAM, Protospacer adjacent motif. AP, apurinic/apyrimidinic sites. Star in magenta indicates the nick generated by nCas9. **b** Schematic diagram of potential pathway for T (or C) editing and outcomes. A glycosylase variant is designed to remove normal T or C, an nCas9-sgRNA complex creates an R-loop at the target site and nicks the non-edited strand, then the generated AP site is repaired by translesion synthesis (TLS) and/or DNA replication, leading to T or C editing. DSB, double-strand break. indel, insertion and deletion. **c** Schematic of various gTBE and gCBE candidate architectures. The bipartite nuclear localization signal (bpNLS) is shown in dark gray, linker in light gray, nCas9 in teal green, and UNG in light green. Note that Y156A (purple line) and N213D (red line) of UNG2 are equivalent to Y147A and N204D of UNG1, respectively. Δ1-88: 1-88 amino acids truncation of UNG2. **d** Percentage of EGFP^+^ cells for T editing activity evaluation of different gTBE variants using T-to-G reporter (*n* = 3 independent biological replicates). NT, non-target sgRNA. T: target sgRNA. **e** Percentage of EGFP^+^ cells for C editing activity evaluation of different gCBE variants using C-to-G reporter (*n* = 3 independent biological replicates). NT, non-target sgRNA. T: target sgRNA. **f** the orthogonality of gTBE and gCBE for base editing evaluated using two different reporters (*n* = 3 independent biological replicates). All values are presented as mean ± s.e.m. Source data are provided as a Source Data file. Panel (**a**) adapted from Tong et al.^3^ (copyright 2023).

Encouraged by the development of gGBE in our previous study^3^, we attempted to develop thymine and cytosine base editor using the deaminase-free glycosylase-based strategy. Since the three pyrimidine bases (i.e., T, C, and U) are structurally similar, we speculated that excision of canonical T or C could be achieved by engineering certain uracil DNA glycosylase (UNG). The excision of T or C would generate apurinic/apyrimidinic (AP) sites, then trigger the base excision repair (BER) pathway and facilitate direct T editing or C editing (Fig. 1a, b). Alternative splicing as well as transcription from two distinct start sites leads to two different human UNG isoforms, the mitochondrial UNG1 (304 amino acids, aa) and the nuclear UNG2 (313 aa), each possessing unique N-termini that mediate translocation to the mitochondria and the nucleus, respectively^16^ (Supplementary Fig. 1). Two human UNG1 variants, UNG1-Y147A and UNG1-N204D, have been engineered to excise T and C in DNA, respectively^17^. Y156A and N213D of UNG2 are equivalent to Y147A and N204D of UNG1, respectively. To edit the nuclear DNA, we generated two prototype gBEs, a deaminase-free glycosylase-based thymine base editor (gTBE) and a deaminase-free glycosylase-based cytosine base editor (gCBE), by fusing UNG2-Y156A and UNG2-N213D at the C-terminus of Cas9 D10A nickase (nCas9), respectively (Fig. 1a, c). We developed T-to-G reporter and C-to-G reporter, two intron-split EGFP reporter systems as reported previously^9^, to evaluate the editing activity of gTBE and gCBE, respectively (Supplementary Fig. 2a). In these reporters, the AG-to-AT or AG-to-AC inactive splicing acceptor (SA) could only be remediated with T-to-G or C-to-G conversion, thus leading to correct splicing of EGFP-coding sequence and EGFP activation (Supplementary Fig. 2b). The gBE vectors were co-transfected with the T-to-G or C-to-G reporter vector containing the single-guide RNA (sgRNA) that targets the corresponding mis-splicing mutations. We found that gTBE with UNG2-Y156A (hereafter referred to as gTBEv0.1) showed slight T-to-G conversion activity, and gCBE with UNG2-N213D (hereafter referred to as gCBEv0.1) showed slight C-to-G conversion activity (Fig. 1c–e).

Given the disordered N-terminal domain (NTD) of UNG contains protein binding motifs and sites for post-translational modifications^18^, which might constrain targeted excision activity of the glycosylase domain in ssDNA^19,20^, we constructed UNG-NTD-truncated gTBE and gCBE versions with UNG2Δ88 (1-88 amino acids truncation of UNG2) variants (Fig. 1c) to eliminate undesired protein-protein interactions^20–22^. The gTBEv0.2 with UNG2Δ88-Y156A fused at the C-terminus exhibited comparable T-to-G conversion activity with gTBEv0.1 (1.0% vs. 1.1%, Fig. 1d), while gCBEv0.2 with UNG2Δ88-N213D fused at the C-terminus increased the C-to-G conversion activity compared with gCBEv0.1 (13.3% vs. 1.0%, Fig. 1e). Moreover, the gTBEv0.3 with UNG2Δ88-Y156A and gCBEv0.3 with UNG2Δ88-N213D fused at the N-terminus showed much higher editing activity than those at the C-terminus (10.2% vs. 1.0%, and 51.4% vs. 13.3%, Fig. 1c-e), a 10- and 3.9-fold enhancement in the editing efficiency, respectively. No editing activity was found for all the above-mentioned versions of gTBE and gCBE together with the non-targeting sgRNA (Fig. 1d, e). In addition, gTBEv0.3 exhibited the highest T-to-G editing activity among various UNG-NTD-truncated versions of gTBE (Supplementary Fig. 3).

Furthermore, we examined the orthogonality of gTBE and gCBE for base editing. Although engineered from the same original glycosylase UNG, no C editing activity was found for gTBEv0.3 and no T editing activity was found for gCBEv0.3 (Fig. 1f). Thus, we developed two orthogonal base editors, gTBE for direct T editing and gCBE for C editing.

### Evolution of gTBE with enhanced editing activity

**Fig. 2:**
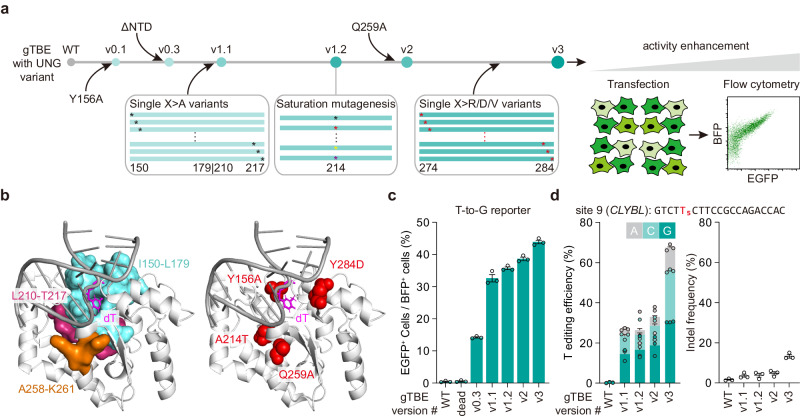
Protein engineering and evolution of gTBEs. **a** Schematic diagram of mutagenesis and screening strategy for the engineered gTBE. The EGFP reporter plasmids were transiently co-transfected into cultured cells along with the gTBE plasmids, and the fluorescence intensity of EGFP was detected with flow cytometry. ΔNTD: N-terminal domain (NTD) truncation of UNG. **b** Left, the selected residues (shown as surface) for mutagenesis nearby the catalytic site pocket of human UNG-DNA complex (PDB entry 1EMH^24^), in which dΨU was mutated to T in the DNA (dT). I150-L179 are highlighted in cyan, L210-T217 in magenta, A258-K261 in orange. Right, location of the effective residues in gTBEv3 variant shown as spheres in red on the three-dimensional structure. **c** Gradual improvement of EGFP activation for each gTBE variants (*n* = 3 independent biological replicates). WT, wild-type UNG2Δ88. dead, catalytically inactive UNG2Δ88 (carrying D154N and H277N mutations, equivalent to D145N and H268N of UNG1)^60^. **d** Frequencies of T base editing outcomes (left) and indels (right) with different gTBE variants at the edited T5 position in site 9 (*CLYBL* gene) in transfected HEK293T cells by target deep sequencing (*n* = 3 independent biological replicates). All values are presented as mean ± s.e.m. Source data are provided as a Source Data file. Panel (**a**) adapted from Tong et al.^3^ (copyright 2023).

To further increase the T-to-G activity of gTBEv0.3, we attempted to perform rational mutagenesis for engineering the UNG moiety, using the T-to-G reporter to evaluate the editing activity in cultured mammalian cells (HEK293T) (Fig. 2a). Based on structural and functional analysis, WT UNG contains five conserved motifs required for efficient glycosylase activity: the catalytic water-activating loop, the proline-rich loop, the uracil-binding motif, the glycine-serine motif and the leucine loop^23–25^ (Supplementary Fig. 1b). Since Y156 in the catalytic water-activating loop and N213 in the uracil-binding motif are critical for activity switch from U excision to T or C excision, we firstly selected sequential and spatial neighbors of these two residues and examined their roles in the regulation of base excision activity (Fig. 2a, b). We conducted alanine-scanning mutagenesis by replacing all non-alanine with alanine (X > A) and alanine with valine (A > V) to cover all the residues in the regions of I150-L179 and L210-T217. Interestingly, we obtained a variant gTBEv1.1 (v0.3 with A214V) largely elevating the T-to-G conversion activity by 2.68-fold (Supplementary Fig. 4a). To check whether there is any amino acid at position 214 performing better than the valine, we further performed site-saturation mutagenesis focused on the residue at position 214. We obtained gTBEv1.2 (v0.3 with A214T) with elevated editing efficiency by 1.06-fold in comparison with the T editing activity of gTBEv1.1 (Supplementary Fig. 4b).

Then, we examined the spatial neighbors of residue T214, nearby the Gly-Ser loop that compresses the DNA backbone 3′ to the lesion (Fig. 2b), and obtained variant gTBEv1.3 (v0.3 with Q259A), which increased the editing efficiency by 1.46-fold (Supplementary Fig. 4c). Furthermore, we found a synergistic enhancement of T-to-G editing activity in variant gTBEv2 (v0.3 with combination of A214T and Q259A), by 2.7-fold in comparison with the T editing activity of gTBEv0.3 (Fig. 2c). We also scanned residues in the regions of Q274-Y284, in or nearby the Leu-intercalation loop, by sequential replacement with amino acids of distinct properties, including arginine (with positive charged side chain), aspartic acid (with negative charged side chain), or valine (with small hydrophobic side chain) (X > R, D, or V). Although most of these mutations reduced the T editing activity, we found a variant gTBEv3 (v2 with Y284D) showed elevated editing efficiency by 1.22-fold as compared with that of gTBEv2 (Supplementary Fig. 5), and by 3.09-fold compared with gTBEv0.3 (Fig. 2c).

We validated the improvement of T editing activity by different gTBE variants at one endogenous genomic site in HEK293T. After transfected with all-in-one constructs encoding each gTBE variant, together with sgRNA that targeted site 9 in *CLYBL* gene and mCherry for fluorescence-activated cell sorting (FACS), mCherry-positive cells were FACS-sorted. Through target deep sequencing analysis, we obtained a gradual increase of overall T editing efficiency at T5 from 26.9% for gTBE1.1 to 67.4% for gTBE3, as well as the insertions and deletions (indels, from 3.6% to 13.3%), with T-to-S (i.e., T-to-C or T-to-G; S = C or G base) conversions as the predominant events at this site (Fig. 2d). These results indicate that rounds of mutagenesis described above had effectively optimized gTBE activity for T-to-C and T-to-G base editing. Thus, the engineered version of gTBEv3 (carrying Y156A, A214T, Q259A, Y284D mutations) had the highest T editing efficiency and was used for the following studies.

### Characterization of gTBEv3 at human genomic DNA sites

**Fig. 3:**
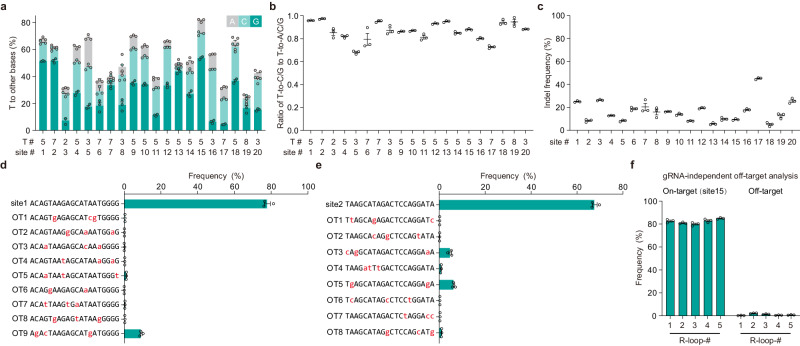
Characterization of editing profiles of gTBE via target deep sequencing. **a** Bar plots showing the on-target DNA base editing at positions with the highest T conversion frequencies at each genomic site in HEK293T cells (mean ± s.e.m., *n* = 3 independent biological replicates). T#: T position with highest on-target base editing frequencies across protospacer positions 1–20. site #: genomic site number. **b** The ratio of T-to-C/G to T-to-A/C/G conversion frequency by gTBEv3 editing at the sites shown in (**a**). **c** Indels frequencies with gTBEv3 at 20 on-target sites (*n* = 3 independent biological replicates). **d**, **e** The sgRNA-dependent off-target analysis for gTBEv3 editing efficiency at site 1 and site 15 (*n* = 3 independent biological replicates). OT: off-target. **f** The sgRNA-independent off-target editing efficiency detected by the orthogonal R-loop assay at each R-loop site (*n* = 3 independent biological replicates). All values are presented as mean ± s.e.m. Source data are provided as a Source Data file.

We further characterized the editing profiles of gTBEv3 by targeting 20 endogenous genomic loci, most of which were used in previous base editing studies^11,12,26,27^. We found that gTBEv3 achieved efficient T base editing activity (ranged from 24.3% to 81.5%; Fig. 3a and Supplementary Fig. 6a, b), but essentially no A, C or G editing at all examined sites (Supplementary Fig. 6c–e). The T-to-C or T-to-G conversions were the predominant events (Supplementary Fig. 6f–h), only a low percentage of T-to-A conversion were detected (Fig. 3a and Supplementary Fig. 6i), consistent with previous findings of gGBE^3^, AYBE^9^ and CGBEs^11–15^. The ratios of T-to-S to T conversion ranged from 0.68 to 0.97 (without indels, Fig. 3b) and from 0.41 to 0.92 (with indels, Supplementary Fig. 6j). We found that gTBEv3 also induced indels with frequency ranging from 5.2% to 45.2% at the 20 edited sites (Fig. 3c). Furthermore, the editable range of gTBEv3 was positions 2 to 11, and the optimal editing window with high efficiency of T conversion covered protospacer positions 3 to 7, with the highest editing efficiency at position 5 (Supplementary Fig. 6b). We found no obvious motif preference for T conversions with gTBEv3 by analyzing the on-target editing and sequences of all tested sites (Supplementary Fig. 6k).

We have analyzed the off-target activity of gTBEv3 at several in silico-predicted^28^ guide-dependent off-target sites, and characterized the ability of gTBEv3 to mediate guide-independent off-target DNA editing using orthogonal R-loop assay in five previously reported dSaCas9 R-loops^9,29^. We found very low percentage of editing at all the guide-dependent off-target loci (Fig. 3d, e and Supplementary Fig. 7) and detected very low frequencies (1.1% in average) at all five guide-independent off-target sites (Fig. 3f). Taken together, the gTBEv3 represents a highly efficient T-to-S base editor with low off-target effects in mammalian cells.

### Enhancement of C editing activity of gCBE

**Fig. 4:**
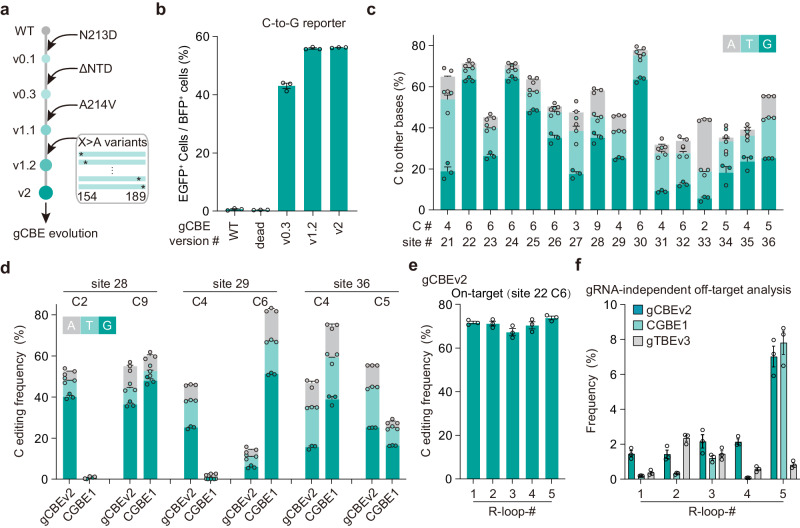
Enhancement of gCBE editing activity through protein engineering. **a** Schematic diagram of mutagenesis and screening strategy for the engineered gCBE. **b** Gradual improvement of EGFP activation for each gCBE variants (*n* = 3 independent biological replicates). WT, wild-type UNG2Δ88. dead, catalytically inactive UNG2Δ88 (carrying D154N and H277N mutations, equivalent to D145N and H268N of UNG1)^60^. ΔNTD: N-terminal domain (NTD) truncation of UNG. **c** Bar plots showing the on-target DNA base editing at positions with the highest C conversion frequencies at each genomic site in HEK293T cells (*n* = 3 independent biological replicates). C#: C position with highest on-target base editing frequencies across protospacer positions 1–20. site #: genomic site number. **d** Bar plots showing the on-target DNA base editing of different positions at three loci with gCBEv2 or CGBE1 (*n* = 3 independent biological replicates). **e** On-target base editing frequencies for gCBEv2 at C6 of site 22 in HEK293T cells for the orthogonal R-loop assay (*n* = 3 independent biological replicates). **f** gRNA-independent cumulative off-target editing frequencies detected by the orthogonal R-loop assay at each R-loop site. Each R-loop was performed by co-transfection of each base editor, and an SpCas9 sgRNA targeting corresponding site with dSaCas9 and a SaCas9 sgRNA (*n* = 3 independent biological replicates). All values are presented as mean ± s.e.m. Source data are provided as a Source Data file.

To examine whether the mutations emerged from the engineering of gTBE would benefit the enhancement of gCBE activity, we attempted to generate gCBEv1.1 by introducing A214V into gCBEv0.3 (Fig. 4a). We found that the gCBEv1.1 largely elevated the C-to-G conversion activity by 1.34-fold when evaluated using the C-to-G reporter (Supplementary Fig. 8a). We conducted alanine-scanning mutagenesis on the fragment of D154-D189 to examine its role in the regulation of base excision activity, and obtained a variant gCBEv1.2 (v0.3 with K184A) largely elevating the C-to-G conversion activity by 1.55-fold (Supplementary Fig. 8b). We further investigated the additive effect of A214V and K184A by combining these two mutations in gCBEv2 (carrying K184A, N213D, A214V mutations), and found synergistic enhancement of C-to-G editing activity by 1.3-fold compared with that of gCBEv0.3 (Fig. 4b). We further validated the improvement of C editing activity for different gCBE variants by targeting an endogenous genomic site, and found a gradual increase of overall C editing efficiency from 18.2% to 37.2% at C2 of the site 28 (Supplementary Fig. 9a).

By targeting 16 endogenous genomic loci, we characterized the editing profiles of gCBEv2 and obtained efficient C base editing activity ranged from 31.8% to 77.7% (Fig. 4c and Supplementary Fig. 9b–d). We found that gCBEv2 could induce predominant C-to-G conversions as well as C-to-T conversions, with the ratios of C-to-G/T to C-to-A/G/T conversion reaching up to 0.97, and there were very few C-to-A conversions detected (Fig. 4c, Supplementary Fig. 9e–h). The gCBEv2 could induce indels with frequency ranged from 3.1% to 48.3% at the examined sites (Supplementary Fig.9i). After analyzing the sequences of all tested sites, we found that the editable range of gCBEv2 was positions 2 to 9 (Supplementary Fig. 9c), and gCBEv2 showed preferences for editing at AC or TC motifs with a higher efficiency than other motifs (Supplementary Fig. 9j).

When compared to CGBE1^12^, a C-to-G base editor, we found that gCBEv2 showed higher editing activity at certain positions towards the distal end of the target sequence (Fig. 4d and Supplementary Fig. 9c), indicating their positional preferences within different optimal editing windows (positions 2 to 6 for gCBEv2 vs. positions 5 to 7 for CGBE1^12^). The gCBEv2 induced fewer indels at site 36, and more indels at site 28 and site 29 than CGBE1 (Supplementary Fig.9k). To be noted, using the orthogonal R-loop assay^9,29^ mentioned above, we found that gCBEv2 showed comparable frequencies with CGBE1 at two guide-independent off-target sites, but higher at the other three sites (Fig. 4e, f and Supplementary Fig. 9l).

Moreover, we found that the gCBEv2 could only facilitate C editing, but there was essentially no T editing at all examined sites (Supplementary Fig. 9c,d). The editing specificity of gCBEv2, together with that of gTBEv3 (Supplementary Fig. 6b–e), consolidated the orthogonality of these two base editors for base editing.

### Applications of gTBE and gCBE

**Fig. 5:**
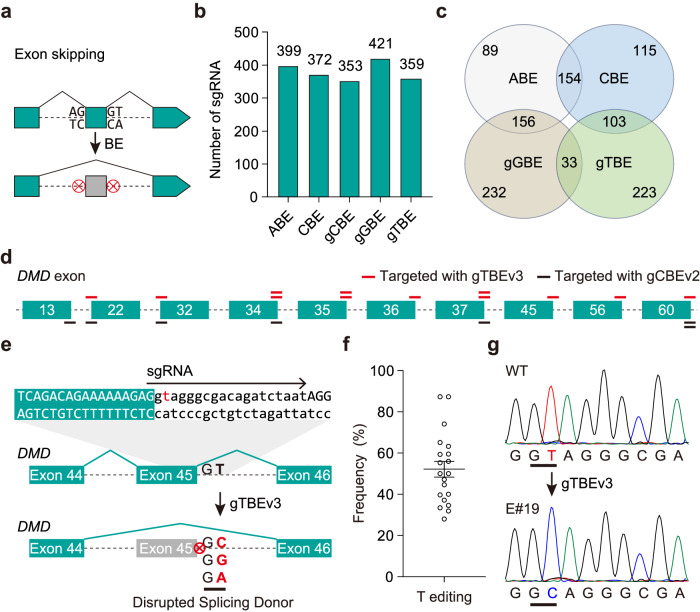
Gene editing applications of gTBE and gCBE. **a** Principle for exon skipping with base editors. **b** Bar plots showing the numbers of sgRNA candidates targeting the splicing sites in 16 genes by different base editors. gCBE, gCBEv2; gGBE, gGBEv6.3; gTBE, gTBEv3. The 16 genes are *AGT, ANGPTL3, APOC3, B2M, CD33, DMD, DNMT3A, HPD, KLKB1, PCSK9, PDCD1, PRDM1, TGFBR2, TRAC, TTR*, and *VEGFA*. **c** Venn diagram showing the distribution of sgRNAs for 4 base editors in (**b**). **d** Schematic diagram illustrating sgRNA candidates specifically targeting SD or SA sites in human *DMD* with gTBEv3 (red lines) or gCBEv2 (black lines), but not ABE or CBE. **e** Schematic diagram illustrating the skipping of human *DMD* exon 45 induced by gTBE-induced disruption of the splicing donor site. **f** On-target base editing efficiency for gTBEv3 targeting the splicing donor site of humanized *DMD* exon 45 in mouse embryos (mean ± s.e.m., *n* = 20). **g** DNA sequencing chromatograms from wild-type (WT) and representative embryos co-injected with gTBEv3 mRNA and sgRNA targeting the SD site of human *DMD* exon 45. Source data are provided as a Source Data file.

We further evaluated the potential applications of gTBE and gCBE. The gTBE could not only remediate inactive splicing signals in the intron-split EGFP reporter systems used above (Figs. 1, 2 and Supplementary Fig. 2), but also be used for exon skipping by disrupting splicing signals at splicing donor (SD) or splicing acceptor (SA) sites (Fig. 5a). After analyzing the splicing sites in 16 well-studied genes for gene and cell therapy research^30–32^, we found that gTBE and gCBE, together with other existing base editors, provide 1904 sgRNA candidates (Supplementary Data 3) with the SD or SA sites located in each optimal editing window (Fig. 5b and Supplementary Fig. 10a). Among the 771 sgRNA candidates for ABE and CBE targeting, 156 and 103 candidates overlapped with those for gGBE and gTBE, respectively (Fig. 5c). Moreover, 232 and 223 sgRNA candidates could only be screened by gGBE or gTBE targeting, respectively (Fig. 5c). For gCBE, apart from 205 sgRNA candidates overlapped with those for CBE, there were 148 unique candidates (Supplementary Fig. 10b). The availability of these base editors could largely expand the scope of sgRNA screening for efficient editing at splicing sites (Supplementary Fig. 10). In addition, the developed base editors could be utilized for bypassing premature termination codons (PTCs) and introduction of PTCs (Supplementary Fig. 11). The gTBE and gCBE could provide more versatile codon outcomes from PTCs editing (Supplementary Fig. 11b), and introduce PTCs by editing more codons coding various amino acids (Supplementary Fig. 11d). To potentially disrupt gene function by introduction of PTCs, we analyzed and obtained 851 sgRNA candidates (Supplementary Data 4) targeting various codons for PTCs introduction in 15 genes with gGBE and CBE, with 191 TACs and 124 TCAs for gGBE targeting (Supplementary Fig. 11e).

To illustrate these applications, we focused on editing the splicing sites in human *DMD* gene (*Duchenne muscular dystrophy*, coding dystrophin) that cannot be targeted with ABE or CBE. We designed and screened a series of sgRNAs specifically targeting SD or SA sites with gTBEv3 or gCBEv2 (Fig. 5d and Supplementary Fig. 10c), including three sgRNAs targeting the SD sites of *DMD* exon 45 (Fig. 5e), 12 and 37 (Supplementary Fig. 10d) uniquely targeted by gTBEv3. Disruption of the SD site of exon 45, thus leading to exon skipping, would be applicable to restore dystrophin expression in 9% DMD patients^33^. Thus, we co-injected gTBEv3 mRNA and sgRNA targeting the SD site of *DMD* exon 45 into zygotes of humanized mice to explore the potential application of gTBE. We found 100% (20/20) mouse embryos harbored efficient base conversion (ranged from 28.0% to 87.4%) at the desired position T3 (Fig. 5f, g), indicating the great potential of gTBE for human disease modeling and gene therapy. Overall, gBEs, including gTBE, gCBE and gGBE, provide more options for the sites that dBEs could not target, largely expanding the targeting scope of base editors.

### Comparison of different editing systems

**Fig. 6:**
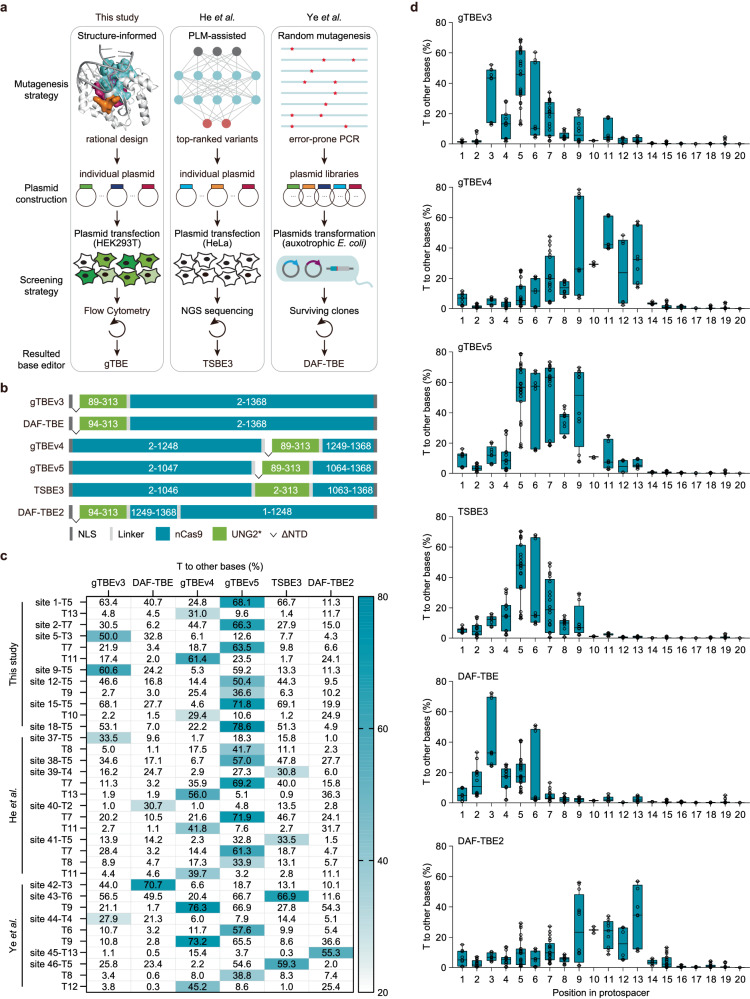
Comparison of different gTBEs. **a** The strategies for protein engineering and screening used in three studies. **b** Schematic of the basic architectures for various base editors. The bipartite nuclear localization signal (bpNLS) is shown in dark gray, linker in light gray, and nCas9 in teal green. UNG2* (in light green), UNG2 variant from the corresponding base editor. ΔNTD, deletion of the N-terminal domain. **c** The frequencies of T conversions at 17 endogenous loci. The thymines with editing frequencies > 25% for any base editors were showed. The highest frequencies at corresponding positions were highlighted as Heat map (*n* = 3 indepe*n*dent biological replicates per site. Note *n* = 2 for site 44 targeted by gTBEv4.). **d** Frequencies of T conversions by various base editors across the protospacer positions 1–20 (where PAM is at positions 21–23) from the edited sites in (**c**). Single dot represents individual replicate, and boxes span the interquartile range (25th to 75th percentile); horizontal lines within the boxes indicate the median (50%); and whiskers extend to the minimal and maximal values. Source data are provided as a Source Data file.

In this study, we have engineered gTBEs and gCBEs using structure-informed rational mutagenesis (Fig. 6a). During the peer review process of this work, two studies reported several independently developed deaminase-free glycosylase-based base editors^34,35^. He et al. developed a TSBE3 for T-to-G/C substitutions using protein language model (PLM)-assisted strategy^34^, while Ye et al. conducted rounds of random mutagenesis by error-prone PCR for directed evolution in *Escherichia coli* and obtained several deaminase-free base editors (DAF-TBEs and DAF-CBEs)^35^ (Fig. 6a). The basic architectures of above-mentioned base editors are different, for instance, TSBE3 was constructed using an embedding strategy and DAF-TBE2 using a circularly permuted strategy (Fig. 6b). Since embedding of deaminase or glycosylase into the Cas9 domain could modulate the editing efficiency and/or editing window of certain base editor^10,36–38^, we generated gTBEv4 and gTBEv5 by inserting the engineered UNG2 variant of gTBEv3 into the nCas9 domain at different locations (Fig. 6b).

To better characterize the performance of various deaminase-free base editors, we made a side-by-side comparison of base editors in our study and those from the other two studies. We first compared the T editing efficiency of various thymine base editors at 17 endogenous sites, including five sites from He’s study^34^ and five sites from Ye’s study^35^ (Fig. 6c and Supplementary Fig. 12). For base editors with UNG variant fused at the N-terminus of nCas9, gTBEv3 showed higher editing efficiency than DAF-TBE at the overwhelming majority of Ts (29 out of 35) of tested sites (Fig. 6c, Supplementary Fig. 12f), indicating that UNG variants generated by rational mutagenesis are superior to those by random mutagenesis in this situation. We also compared gTBEv3 with gTBEv4 and gTBEv5, two base editors constructed using the embedding strategy. The gTBEv4 showed a shifted editing window of positions 7–13 from positions 3–7 (Fig. 6d), with no significant difference in the average editing efficiency for gTBEv3 (23.2% vs. 23.1%, Supplementary Fig. 12f). For gTBEv5, the editing efficiency was largely increased compared to that of gTBEv3 (averaging 39.3% vs. 23.1%, Supplementary Fig. 12f), with the same predominant T-to-S conversions (Supplementary Fig. 12a–d, g), and the optimal editing window covered protospacer positions 5 to 9 (Fig. 6d). TSBE3 (carrying L83Q and G116E mutations, equivalent to L74Q and G107E in UNG1) is an nCas9-embedded base editor with almost the same insertion position as gTBEv5 (Fig. 6c). The gTBEv5 showed higher editing efficiency than TSBE3 (39.3% vs. 22.5%, Supplementary Fig. 12f) at the overwhelming majority of Ts (29 out of 35) of tested sites (Fig. 6c), indicating that UNG variants generated by rational mutagenesis are superior to those generated by PLM-assisted mutagenesis in this situation. The optimal editing window of TSBE3 covered protospacer positions 4 to 9 (Fig. 6d). The circularly permuted DAF-TBE2 showed an editing window of positions 9–13, different from the editing window (positions 2–6) of DAF-TBE (Fig. 6d). Despite showing the highest average editing efficiency, gTBEv5 induced comparable indel rates to that of DAF-TBE (14.4% vs. 14.4%), DAF-TBE2 (14.4% vs. 10.3%) and TSBE3 (14.4% vs. 13.5%, Supplementary Fig. 12e–g). To be noted, gTBEs induced much fewer unintended T editing than TSBE3 and DAF-TBEs in the proximal DNA sequence upstream from two sites (site 38 and site 44) harboring unintended edits (Supplementary Fig. 13), consistent with the finding that the NTD of UNG could promote targeting the enzyme to ssDNA–dsDNA junctions^19^.

Similarly, we then compared the C editing efficiency of various base editors (Supplementary Fig. 14a) at 19 endogenous sites, including five sites from He’s study^34^ and five sites from Ye’s study^35^ (Supplementary Fig. 14b). We found that gCBEs showed higher overall average editing efficiency than all other base editors (Supplementary Fig. 14b, e). The gCBEv2 outperformed DAF-CBE (30.1% vs. 21.3%) and CGBE-CDG (30.1% vs. 19.3%) for the average efficiency of base conversion (Supplementary Fig. 14c, f), indicating that UNG variants generated by rational mutagenesis are superior to those by random mutagenesis in this situation. Although CGBE1 induced the least indels and gCBEv3 induced more indels, gCBEv2 induced comparable average indel rates with other deaminase-free base editors, including DAF-CBE (16.8% vs. 16.9%), DAF-CBE2 (16.8% vs. 12.1%) and CGBE-CDG (16.8% vs. 13.6%, Supplementary Fig. 14d, g). The C-to-G editing frequency and purity of different base editors show respective advantages for CGBE1 and various deaminase-free base editors at different cytosine position across the protospacer (Supplementary Fig. 15a, b). Each base editor can edit its target base within a certain editable window, that is, positions 2 to 9 for gCBEv2, positions 2 to 11 for gCBEv3, positions 4 to 10 for CGBE1, positions 2 to 9 for CGBE-CDG, positions 2 to 9 for DAF-CBE, and positions 9 to 12 for DAF-CBE2 (Supplementary Fig. 15c).

After analyzing the off-target effects both at some sgRNA-dependent and sgRNA-independent off-target sites, we found that gTBEs and gCBEs induced comparable low-level off-target edits similar to that of other base editors at most sites (Supplementary Fig. 16a–c). Moreover, by performing transcriptome-wide RNA analysis, we found that gTBEv5 and gCBEv3 did not exhibit significant off-target RNA editing or impact the cell’s inherent DNA repair processes (Supplementary Fig. 16d, Supplementary Data 5), consistent with those of DAF-TBE, DAF-CBE, CGBE-CDG and TSBE3^34,35^.

Prime editing (PE) system could theoretically mediate all types of base substitution, including T-to-G conversion and C-to-G conversion^39^. We compared gTBEv3 and gTBEv5 with the recently evolved PE6d system^40^ at six previously reported endogenous sites^35^ in HEK293T cells. The gTBEv3 and gTBEv5 outperformed PE6d or PE6d max for T-to-G conversion at four tested sites, whereas PEs exhibited higher efficiency and purity than gTBEs at the other two sites (Supplementary Fig. 17a, Supplementary Data 6). The gCBEv2 and gCBEv3 outperformed PE6d or PE6d max for C-to-G conversion at five tested sites, whereas PEs exhibited higher efficiency and purity than gCBEv2 at the other one site (Supplementary Fig. 17b, Supplementary Data 6). These findings indicate that base editing and prime editing offer complementary strengths, and base editors generally show more efficient editing if the target base is positioned optimally. In addition, gTBEs and gCBEs also exhibited efficient T and C editing activity across three different human cell lines (HEK293T, U2OS and Huh-7 cells), with slight perturbations of the product purity for gTBEs and comparable substitution frequency of certain base for gCBEs in different cell lines (Supplementary Fig. 18).

Taken together, we found that gTBEs and gCBEs in our study outperformed other base editors, including DAF-TBEs, DAF-CBE, TSBE3 and CGBE-CDG from the other two studies. And the alternative editing windows of different base editors would provide more choices for proper base conversion.

## Discussion

The deaminase-based base editor (dBE) and deaminase-free glycosylase-based base editor (gBE) are currently two main categories of DNA base editors^3^, enabling direct editing of adenine (A), cytosine (C), or guanine (G), but not thymine (T). In human, about 19% of the pathogenic single nucleotide polymorphism (SNP) could be corrected through T-to-G conversion^9^. In this study, we engineered two orthogonal base editors, gTBE and gCBE, that could achieve highly efficient T and C editing in both cultured human cells and mouse embryos. The gTBE and gCBE could greatly broaden the targeting scope of base editors by breaking the limitations of PAM and narrow editing window, thus increasing the opportunity to obtain an efficient strategy for further research. The T-to-S conversion ability of gTBE allows for a variety of gene-editing applications, including editing splicing sites, as well as editing that bypass PTCs.

We have shown that the same original DNA glycosylase could be engineered into enzymes that selectively excise specific nucleotide bases and harnessed to develop base editors using the deaminase-free glycosylase-based strategy. The enhanced editing efficiency could be attributed to mutations in the UNG moiety that facilitate its specific substrate preference or ssDNA-binding activity, or both, which needs to be elucidated by biochemical and structural experiments in the future. The high editing efficiency of gTBEv5 indicates that insertion of UNG variants into nCas9 might enhance the target DNA accessibility by modulate the interaction between the UNG moiety and the target DNA. Although our mutagenesis and screening strategy based on rational design was effective, the mutagenesis was far from saturating the potential mutant repertoire. More other mutations in other positions of UNG would be identified to enhance the editing activity of gTBE and gCBE.

To date, numerous mutagenesis strategies for protein engineering have been reported, including structure-informed rational mutagenesis, random mutagenesis, and PLM-assisted mutagenesis. However, researchers are very concerned about the selection of a suitable mutagenesis strategy. The three above-mentioned mutagenesis strategies have been independently applied to develop deaminase-free glycosylase-based base editors with similar function by engineering the same original uracil DNA glycosylase variant. We used structure-informed rational design and successfully engineered gTBE and gCBE enabling highly efficient T and C editing, respectively. He et al. utilized PLM to assist the engineering of TSBE3, while Ye et al. obtained DAF-TBE and DAF-CBE by performing random mutagenesis (Fig. 6a). In this study, we systematically compared the glycosylase-mediated base editors developed in different studies. We found that gTBE/gCBE in our study outperformed DAF-TBE, DAF-CBE, TSBE3 and CGBE-CDG, with higher average editing efficiency and alternative editing windows (Fig. 6c, d and Supplementary Figs. 14, 15). Therefore, UNG variants generated by structure-informed rational mutagenesis are superior to those generated by PLM-assisted mutagenesis and random mutagenesis in this situation.

Although we have evaluated the off-target effects of gTBE and gCBE on several targeted genes, a comprehensive analysis through high-throughput whole-genome sequencing methods, such as GOTI^41^ and SAFETI^42^, is required for a thorough assessment of off-target effects before their potential therapeutic applications. Wild-type UNG proteins are highly specific against uracil in both ssDNA and dsDNA, with a preference for ssDNA^43^. The NTD of UNG containing motifs and sites for undesired protein-protein interactions and post-translational modifications could promote targeting the enzyme to ssDNA–dsDNA junctions^19,20^. TSBE3, with full length UNG2, and DAF-TBEs induced more undesired edits than gTBEs in the proximal DNA sequence upstream from two sites harboring unintended edits (Supplementary Fig. 13). Despite wide editable windows and undesired edits with the current gTBEs and gCBEs (Fig. 6d and Supplementary Fig. 14c), a more accurate gTBE or gCBE with a refined editing window might be achieved through further engineering of the glycosylase moiety or architectures of these base editors, encouraged by the development of ABE9^44^ or YE1-BE3^45^.

We note that indels induced by gTBE and gCBE, as well as by AYBE, AXBE and CGBEs that generating AP sites, were higher than those by ABE or CBE^4,5,9–15^. AYBE and CGBE facilitate base editing following a two-step generation of AP sites, while gTBE and gCBE facilitate direct T editing or C editing following the one-step generation of AP sites. Encouraged by the previous studies on CGBE^12,15^ and AYBE^9^, additional effort is required to further reduce the level of off-target editing or indels through engineering approaches. During the development of AYBEv3 by combining the mutations in AYBEv1 and AYBEv2, the indel frequency was synergistically reduced at the VISTA enhancer site^9^. Recently, two studies showed that the suicide enzyme HMCES could reduce the indel byproducts induced by the glycosylase-mediated CGBE^46^ and TSBEs^34^. Yuan et al. have developed eOPTI-CGBE and cOPTI-CGBE with the *E. coli* or *C. elegans* UNG (eUNG or cUNG), respectively, achieving high C-to-G transversion efficiency with low off-target effects^15^.

Moreover, there is need for understanding substitution frequency variations across more cell/tissue types in the future. More specific T-to-C, T-to-G, or C-to G editors could potentially be achieved by harnessing the DNA repair machinery in the BER pathway^9,47–51^ or by further structural fine-tuning of gTBE or gCBE. Several studies have reported that fusion or co-expression of specific translesion synthesis (TLS) polymerase preferentially incorporating certain base opposite AP sites would increase the certainty of base editing outcomes^9,13,49^.

In summary, we have engineered two orthogonal base editors based on the same original DNA glycosylase for direct T editing and C editing, and structure-informed rational design represents an efficient and efficacious protein engineering strategy, providing reference and solving thought for the subsequent evolution of other proteins.

## Methods

### Ethical statement

This research complies with all relevant ethical regulations; the Biomedical Research Ethics Committee of Center for HuidaGene Therapeutics Co. Ltd. approved the study protocol.

### Molecular cloning

Base editor constructs used in this study were cloned into a mammalian expression plasmid backbone under the control of a EF1α promoter by standard molecular cloning techniques, and the two intron-split EGFP reporters were constructed similar to those described previously^9^, except that the engineered sequence containing the last 86 base pairs (bp) intron of human *RPS5* gene was inserted between BFP and EGFP coding sequences. And the corresponding mutations at the splice acceptor site were made to construct T-to-G reporter or C-to-G reporter via site-directed mutagenesis by PCR, respectively. Mutations at the splice acceptor site led to inactive EGFP production. Encouraged by the findings from previous base editors^12,15^, the corresponding mutations at the splice acceptor site were put at position 6 across the protospacer. KOD-Plus-Neo DNA polymerase (KOD-401, Toyobo) was used to amplify the insertion fragments, and NEBuilder HiFi DNA Assembly Master Mix (E2621L, New England Biolabs) was used to perform the Gibson assembly of multiple DNA fragments. The Gibson reaction was then transformed into chemically competent *Escherichia coli* DH5α.

The wild-type UNG2 sequence (313 amino acids long) was PCR-amplified from cDNA of HEK293T, UNG2-Y156A, UNG2-N213D, UNG-NTD-truncated mutants and corresponding combinations were constructed via site-directed mutagenesis by PCR. UNG variants were fused at different orientations with respect to nCas9 via Gibson Assembly method. PE6d architecture harbored a human codon-optimized RNaseH-truncated evolved and engineered M-MLV variant with R221K/N394K/H840A mutations in SpCas9. The nick sgRNA and epegRNA with tevoPreQ_1_ motif were cloned into PE6d construct using Golden Gate assembly, resulting in an all-in-one plasmid. For PE6d max, the codon-optimized hMLH1dn was co-expressed with PE6d.

UNG mutagenesis libraries were designed and generated as previously described^52^ with some modification. In brief, the region of 98–313 aa in UNG2 were divided into 8 aa long segments. BpiI-harboring mutants containing Y156A or N213D were introduced via site-directed mutagenesis by PCR. For evolution of gTBE, regions of I150-L179, A158-K261, L210-T217, and Q274-Y284 were selected for rounds of sequential alanine/arginine/aspartic acid/valine substitutions (X > A, R, D, or V). And site-saturation mutagenesis of the residue 214 were conducted to check whether there is any amino acid at this position performing better than the valine. For evolution of gCBE, region of D154-D189 was selected for sequential alanine substitutions (X > A). To cover all the residues in the corresponding segments for sequential alanine substitutions, we mutated alanine to valine (A > V). Oligos coding for the mutants annealed and ligated into corresponding BpiI (Catalog# FD1014, Thermo Fisher) -digested backbone vectors.

The Cas-OFFinder^28^ was used to search for potential guide-dependent off-target sites of Cas9 RNA-guided endonucleases with a maximum of 3 mismatches (with no bulges). For sgRNAs targeting *DMD* splicing sites with an NGN PAM, a PAM-flexible Cas9 variant SpG was used. The sgRNA oligos were annealed and ligated into BpiI sites. The amino-acid sequence for gTBEv3 and gTBEv5 were supplied in Supplementary Table 1. The UNG mutants and corresponding codon substitutions used were listed in Supplementary Data 1.

### Cell culture, Transfection, and flow cytometry analysis

HEK293T (Catalog# BNCC353535, BNCC), Huh-7 (Catalog# BNCC337690, BNCC) and U2OS (Catalog# BNCC352039, BNCC) cells were cultured with DMEM (Catalog# 11995065, Gibco) supplemented with 10% fetal bovine serum (Catalog# 04-001-1ACS, BI) and 0.1 mM non-essential amino acids (Catalog# 11140-050, Gibco) in an incubator at 37 °C with 5% CO_2_.

Mutant screening was conducted in 48-well plates, with 3 × 10^4^ HEK293T cells per well plated in 250 μL of complete growth medium the day before transfection. Between 16 and 24 h after seeding, cells were co-transfected with 250 ng gTBE (or gCBE) plasmids, 250 ng T-to-G (or C-to-G) reporter plasmids and 1 μg Polyethylenimine (PEI) (DNA/PEI ratio of 1:2) per well. For cell transfection of HEK293T, Huh-7 and U2OS for FACS, 5 × 10^5^ cells per well were plated in 12-well plates with 1 ml complete growth medium the day before transfection. After 14–16 h, 2 μg all-in-one plasmids containing gTBE or gCBE and corresponding sgRNA were transfected into cells using PEI (DNA/PEI ratio of 1:2) or FuGENE HD transfection reagent (DNA:FuGENE ratio of 1:3; E2311, Promega). Orthogonal R-loop assays were performed as described previously^9,29^. In brief, 1 μg of gTBE or gCBE plasmid with sgRNA targeting the corresponding site (with mCherry as reporter) and 1 μg of dSaCas9 plasmid with corresponding sgRNA targeting five off-target sites to generate R-loops (with EGFP as reporter) were co-transfected into HEK293T cells using PEI (DNA/PEI ratio of 1:2). For prime editing, 2 μg all-in-one plasmids containing PE6d, nick sgRNA and epegRNA, or 1 μg all-in-one plasmid and 1 μg of hMLH1dn plasmid were co-transfected into cells using PEI (DNA/PEI ratio of 1:2).

At 48 h post-transfection, expression of mCherry, BFP and EGFP fluorescence were analyzed by BD FACS Aria III or Beckman CytoFLEX S. Flow cytometry results were analyzed with FlowJo V10.5.3. The gating strategy in the identification of mCherry^+^, BFP^+^ and EGFP^+^ cells for on-target editing efficiency evaluation was supplied in Supplementary Fig. 2b.

### Target sequencing of endogenous sites and data analysis

Endogenous target sites of interest were amplified from genomic DNA as previously described^9^. Briefly, 10,000 positive cells with mCherry were isolated by FACS after 72 h of transfection, then genomic DNA was extracted and the regions of interest for target sites were amplified by PCR using site-specific primers. The purified PCR products were analyzed by Sanger sequencing (Genewiz).

Target sequencing data analysis was described in the previous paper^3^. In brief, the amplicons were ligated to adapters and sequencing was performed on the Illumina MiSeq platforms, then the targeted amplicon sequencing reads were processed using fastp with default parameters^53^, and further amplicon sequencing analysis were performed by CRISPResso2^54^. T-to-G purity was calculated as T-to-G yield ÷ T-to-other bases (C, G and A) yield. T-to-S conversion ratio was calculated as T-to-S (C and G) yield ÷ T-to-other bases (C, G and A) yield. Protospacer sequences and site-specific primers used for each genomic locus are listed in Supplementary Data 2.

### In *vitro* transcription of gTBEv3 mRNA and *DMD* sgRNA

The mRNA and sgRNA preparations were performed as previously described^3^. In brief, the gTBEv3 plasmids were linearized by the FastDigest KpnI restriction enzyme (Catalog# FD0524, Thermo Fisher), purified using Gel Extraction Kit (Catalog# D2500-03, Omega), and used as the template for in vitro transcription (IVT) using the mMESSAGE mMACHINE T7 Ultra kit (Catalog# AM1345, Thermo Ambion). For *DMD*-sgRNA preparation, we added the T7 promoter sequence to the sgRNA template by PCR amplification. The T7-*DMD*-sgRNA PCR product was purified using Gel Extraction Kit (Catalog# D2500-03, Omega) and used as the template for IVT of sgRNAs using the MEGAshortscript T7 kit (Catalog# AM1354, invitrogen). The gTBEv3 mRNA and *DMD*-sgRNA were purified using the MEGAclear kit (Catalog# AM1908, invitrogen), eluted in RNase-free water and stored at −80 °C until use.

### Animals and microinjection of mouse zygotes

Experiments involving mice were approved by the Biomedical Research Ethics Committee of Center for HuidaGene Therapeutics Co. Ltd. Mice were maintained in a specific pathogen-free facility under a 12-hour dark–light cycle, and constant temperature (20–26 °C) and humidity (40–60%) maintenance.

Super ovulated humanized *DMD* females with human *DMD* exon 45 in C57BL/6 background (4 weeks old) were mated with C57BL/6 males (8 weeks old), and females from the ICR strain were used as foster mothers. Fertilized embryos were collected from oviducts 21 h post hCG injection. For zygote injection, the mixture of gTBEv3 mRNA (250 ng/µL) and *DMD*-sgRNA (100 ng/µL) was injected into the cytoplasm of 1-cell embryo in a droplet of M2 medium using a FemtoJet microinjector (Eppendorf) with constant flow settings. The injected embryos were cultured in M16 medium with amino acids to blastocysts for three days (37 °C and 5% CO_2_) before genomic DNA extraction and target amplification.

### RNA sequencing experiments

HEK293T cells were plated in 12-well plates as above and transfected with 2 μg of gTBEv5, gCBEv3, CGBE1 or mCherry plasmids using PEI (DNA/PEI ratio of 1:2). At 48 hours after transfection, around 5 × 10^6^ cells were collected. Total RNA was extracted with a TRIzol-based method, fragmented and reverse transcribed to cDNAs with HiScript Q RT SuperMix according to the manufacturer’s instructions. Total RNA integrity was quantified using an Agilent 2100 Bioanalyzer. The RNA-seq library was qualified using the Illumina NovaSeq 6000 platform (performed by GENEWIZ). Trimmomatic (v.0.39-2)^55^ were used to filter the RNAseq raw data. The clean reads were aligned to the hg38 reference genome with Hisat2 (v.2.2.1)^56^. RNA editing sites were calculated using REDItools2^57^ with default parameters. The dbSNP (v.146) database downloaded from NCBI was used to filter the sites overlapped with common single nucleotide variants (SNVs). The sites with less than five mutated or nonmutated reads were further filtered.

StringTie^58^ were used to calculate the expression value. DESeq2^59^ were used to calculate differentially expressed genes with FDR < 0.05 and Fold change > 1.

### Statistics & reproducibility

No statistical method was used to predetermine sample size. No data were excluded from the analyses. The experiments were not randomized; The Investigators were not blinded to allocation during experiments and outcome assessment. Experiments were conducted with three independent biological replicates unless otherwise stated in the figure legend. Statistical tests performed by Graphpad Prism 8 included the two-tailed unpaired two-sample *t*-test or Dunnett’s multiple comparisons test after one-way ANOVA.

### Reporting summary

Further information on research design is available in the Nature Portfolio Reporting Summary linked to this article.

### Supplementary information

Supplementary information

Peer Review File

Description of Additional Supplementary Files

Supplementary Data 1

Supplementary Data 2

Supplementary Data 3

Supplementary Data 4

Supplementary Data 5

Supplementary Data 6

Reporting Summary

### Source data

Source Data

## Data Availability

Expression plasmids used in this study have been deposited at Addgene and are available at https://www.addgene.org/Huawei_Tong/ (Addgene plasmid nos. 220617–220621). All data supporting the findings of this study are available in the main text or supplementary information files. The high-throughput sequencing data generated in this study have been deposited in the National Center for Biotechnology Information Sequence Read Archive under BioProject “PRJNA1105444”. The published structure of human UNG-DNA complex is available in the Protein Data Bank (1EMH). Source data are provided with this paper.

## Code availability

Custom scripts for CRISPResso analyses are available on GitHub at https://github.com/yszhou2016/TBE.
