# Supplementary material for: Development of deaminase-free T-to-S base editor and C-to-G base editor by engineered human uracil DNA glycosylase
Source: Nat Commun. 2024 Jun 8;15:4897. doi: 10.1038/s41467-024-49343-5 (PMC11162499; doi:10.1038/s41467-024-49343-5)
Supplement: Supplementary file 1 — Supplementary information [file 41467_2024_49343_MOESM1_ESM.pdf]

Supplementary information

Supplementary Figures

Supplementary Figure 1. Characteristic sequences and motifs of human UNG1 and UNG2.

Supplementary Figure 2. Characterizations of T-to-G and C-to-G reporter system.

Supplementary Figure 3. Editing activity of gTBE and gCBE candidates with various UNG-NTD truncations.

Supplementary Figure 4. Performance of engineered variants in the gTBEv0.3 background.

Supplementary Figure 5. Performance of engineered variants in the gTBEv2 background.

Supplementary Figure 6. Further characterization of editing profiles for gTBEv3.

Supplementary Figure 7. The sgRNA-dependent off-target analysis for gTBEv3 at more sites.

Supplementary Figure 8. Performance of engineered variants in the gCBEv0.3 background.

Supplementary Figure 9. Further characterization of editing profiles for gCBEv2.

Supplementary Figure 10. Base editing at spicing sites with gTBEv3.

Supplementary Figure 11. PTCs editing and introduction for various base editors.

Supplementary Figure 12. Additional comparison of different gTBEs.

Supplementary Figure 13. T editing in the dsDNA upstream from the target site.

Supplementary Figure 14. Comparison of various glycosylase-based base editors for cytosine editing.

Supplementary Figure 15. Additional comparison of various glycosylase-based base editors for cytosine editing.

Supplementary Figure 16. Off-target analysis of various glycosylase-based base editors.

Supplementary Figure 17. Comparison between gTBEs or gCBEs and PEs.

Supplementary Figure 18. Characterization of editing profiles for gTBEs or gCBEs in HEK293T, Huh-7, and U2OS cells.

Supplementary Tables

Supplementary Table 1. Protein sequence of gTBEv3 and gTBEv5 editor.

Supplementary Figures

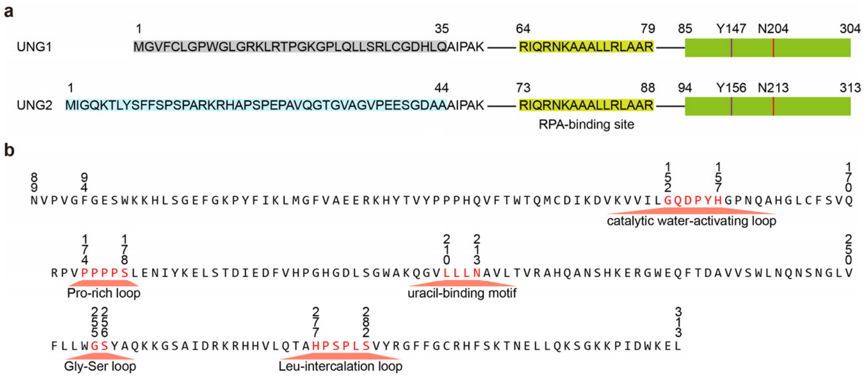

Supplementary Figure 1. Characteristic sequences and motifs of human UNG1 and UNG2. **a**, UNG1-specific N-terminal residues (amino acid 1-35) are marked in grey. UNG2-specific N-terminal residues (amino acid 1-44) are light blue. The common RPA-binding site (yellow) and the globular catalytic domain (light green) are indicated. RPA, Replication protein A. **b**, UNGs contain five conserved motifs numbered from UNG2 as follows: the catalytic water-activating loop (152-GQDPYH-157); the proline (Pro) -rich loop compressing the DNA backbone 5’ to the lesion (174-PPPPS-178); the uracil-binding motif (210-LLLN-213); the glycine-serine (Gly-Ser) loop that compresses the DNA backbone 3’ to the lesion (255-GS-256); and the leucine (Leu) -intercalation loop penetrating the minor groove (277-HPSPLS-282).

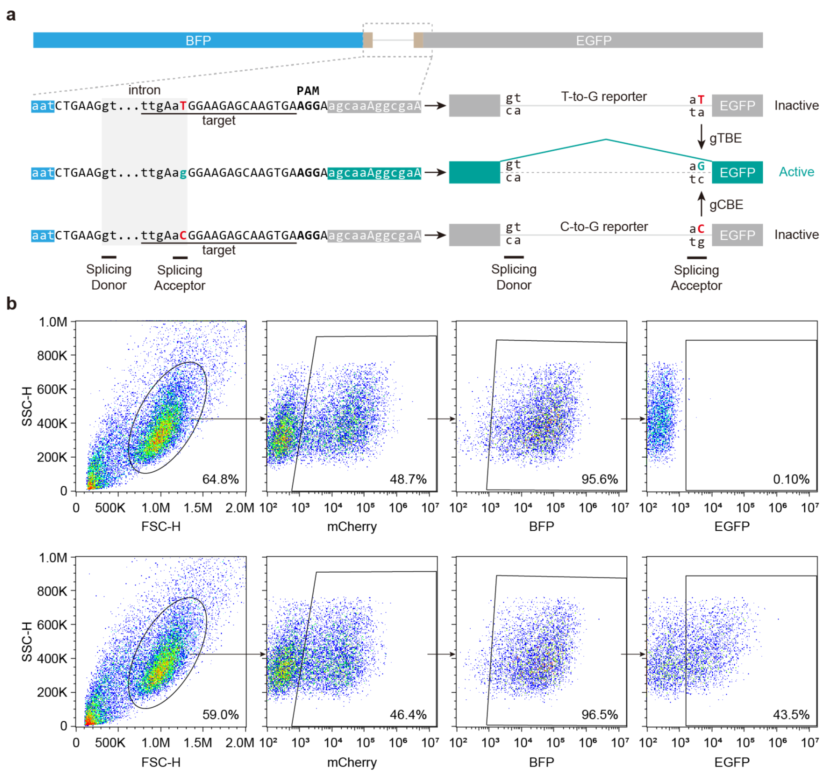

Supplementary Figure 2. Characterizations of T-to-G and C-to-G reporter system.

a, Schematic construct designs of the reporter for T-to-G or C-to-G editing detection. PAM, Protospacer adjacent motif. **b**, Representative flow cytometry scatter plots showing gating strategy and the percentages of gated cells for the negative control (upper panel) and gCBEv0.3 (lower panel).

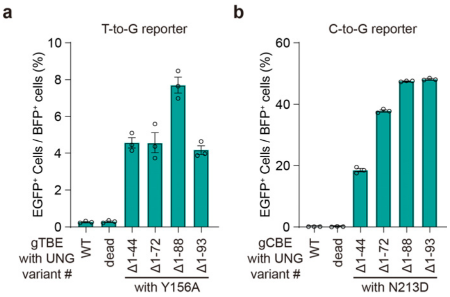

Supplementary Figure 3. Editing activity of gTBE and gCBE candidates with various UNG-NTD truncations.

a, Percentage of $EGFP^+$ cells for evaluating T editing activity of gTBE candidates with various UNG variants (n = 3 independent biological replicates). **b**, Percentage of $EGFP^+$ cells for evaluating C editing activity of gCBE candidates with various UNG variants (n = 3 independent biological replicates). WT, wild-type UNG2Δ88. dead, catalytically inactive UNG2Δ88 (carrying D154N and H277N mutations, equivalent to D145N and H268N of UNG1). All values are presented as mean ± s.e.m. Source data are provided as a Source Data file.

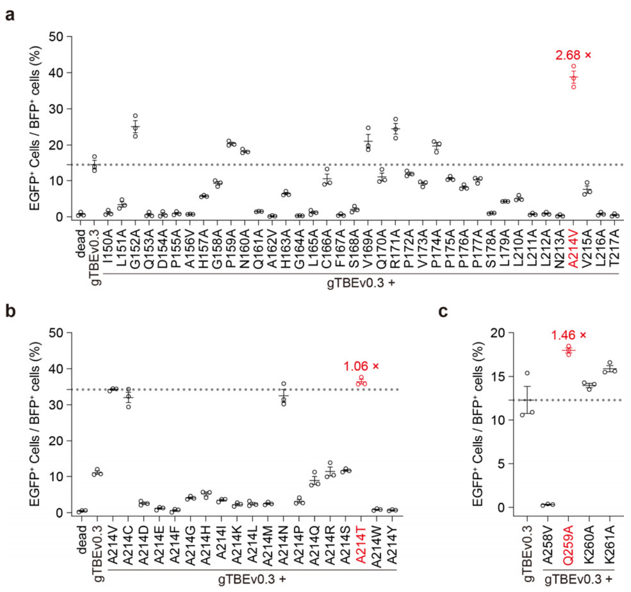

Supplementary Figure 4. Performance of engineered variants in the gTBEv0.3 background.

a, Percentage of EGFP$^+$ cells of gTBE variants from alanine-scanning mutagenesis of regions covering the catalytic water-activating loop, the Pro-rich loop, and the uracil-binding motif (n = 3 independent biological replicates). Replacement of alanine with valine, A>V, is intended to cover all the residues in the interested regions. **b**, Percentage of EGFP$^+$ cells of gTBE variants from site-saturation mutagenesis of the residue at position 214 (n = 3 independent biological replicates). **c**, Percentage of EGFP$^+$ cells of gTBE variants with mutations of selected spatial neighbors of residue T214 (n = 3 independent biological replicates). dead, catalytically inactive UNG2Δ88 (carrying D154N and H277N mutations, equivalent to D145N and H268N of UNG1). All values are presented as mean ± s.e.m. Source data are provided as a Source Data file.

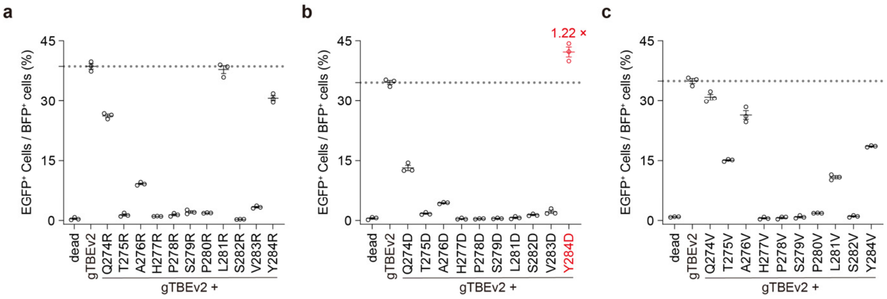

Supplementary Figure 5. Performance of engineered variants in the gTBEv2 background. **a-c**, Percentage of $EGFP^+$ cells of gTBE candidates with different UNG variants from sequential substitutions of arginine (**a**), aspartic acid (**b**), and valine (**c**) (X>R, D, or V) (n = 3 independent biological replicates). dead, catalytically inactive UNG2Δ88 (carrying D154N and H277N mutations, equivalent to D145N and H268N of UNG1). All values are presented as mean ± s.e.m. Source data are provided as a Source Data file.

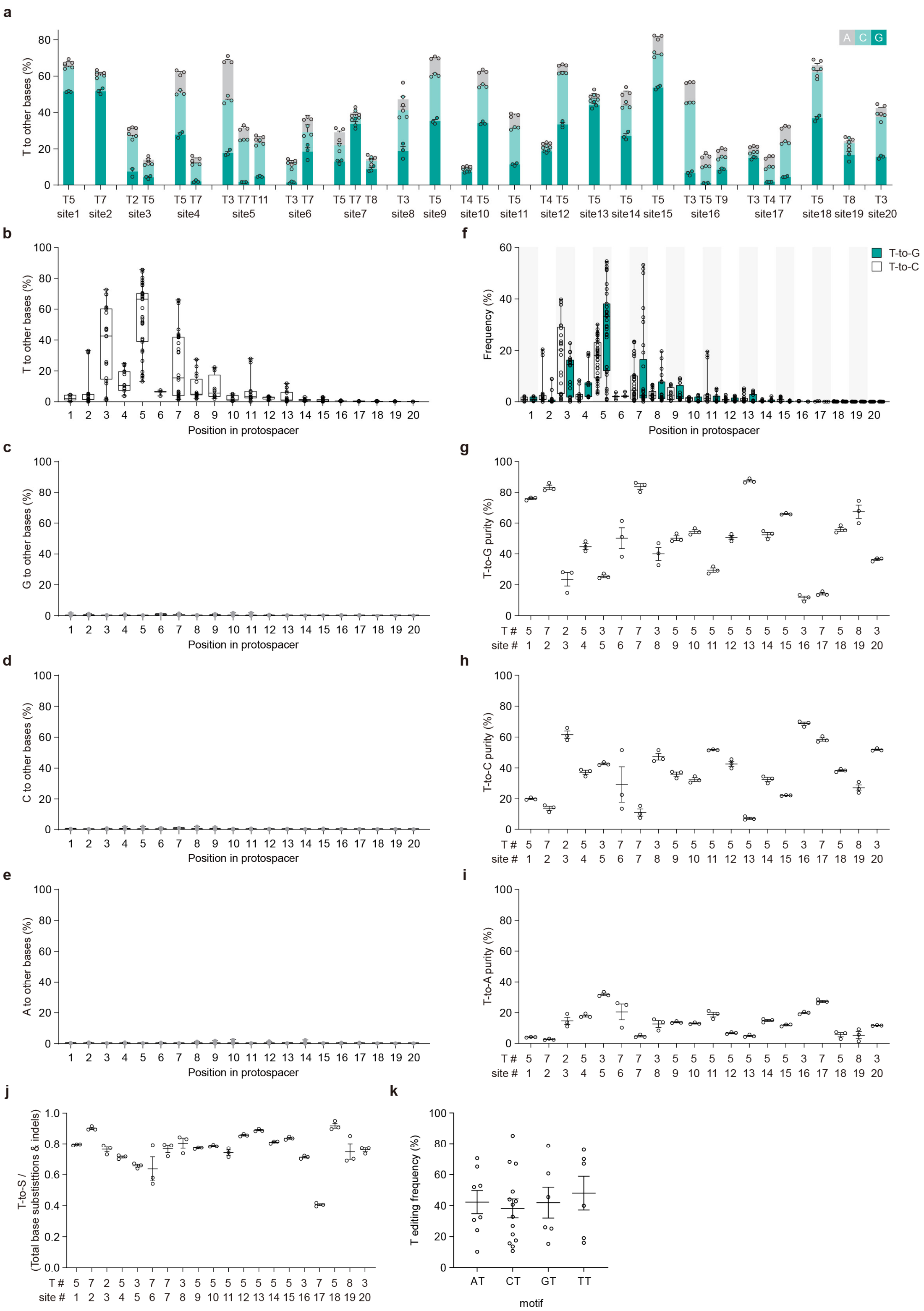

a
T to other bases (%)
A
C
G
T5 site1
T7 site2
T2 T5 site3
T5 T7 site4
T3 T7 T11 site5
T3 T7 site6
T5 T7 T8 site7
T3 site8
T5 site9
T4 T5 site10
T5 site11
T4 T5 site12
T5 site13
T5 site14
T5 site15
T3 T5 T9 site16
T3 T4 T7 site17
T5 site18
T8 site19
T3 site20
b
T to other bases (%)
Position in protospacer
c
G to other bases (%)
Position in protospacer
d
C to other bases (%)
Position in protospacer
e
A to other bases (%)
Position in protospacer
f
Frequency (%)
T-to-G
T-to-C
Position in protospacer
g
T-to-G purity (%)
T #
site #
h
T-to-C purity (%)
T #
site #
i
T-to-A purity (%)
T #
site #
j
T-to-S / (Total base substitutions & indels)
T #
site #
k
T editing frequency (%)
AT
CT
GT
TT
motif

Supplementary Figure 6. Further characterization of editing profiles for gTBEv3.
a, Stacked bar plots showing the on-target DNA base editing at positions with T conversion frequencies >10% at each genomic site in HEK293T cells (mean ± s.e.m., $n = 3$ independent biological replicates). **b-e**, Frequencies of T (**b**), G (**c**), C (**d**) and A (**e**) conversions by gTBEv3 across the protospacer positions 1-20 (where PAM is at positions 21–23) from the edited sites in Figure 3a. **f**, Frequencies of T-to-G and T-to-C editing by gTBEv3. In **b-f**, single dot represents individual replicate ($n = 3$ independent replicates per site), and boxes span the interquartile range (25th to 75th percentile); horizontal lines within the boxes indicate the median (50%); and whiskers extend to the minimal and maximal values. **g-i**, Percentage of T-to-G (**g**), T-to-C (**h**) or T-to-A (**i**) editing by gTBEv3 at various edited sites shown in Figure 3a (mean ± s.e.m., $n = 3$ independent biological replicates). T#: T position with highest on-target base editing frequencies across protospacer positions 1–20. site #: genomic site number. **j**, The ratio of T-to-S to total T editing (base conversions and indels) by gTBEv3 editing at the sites shown in Figure 3a. **k**, The statistical analysis of on-target DNA base editing for each NT motif from the edited sites in (**a**). Each dot represents the mean of three biological replicates for each edited position at various edited sites. $n = 8, 14, 6, 6$ for motif AT, CT, GT, TT. Source data are provided as a Source Data file.

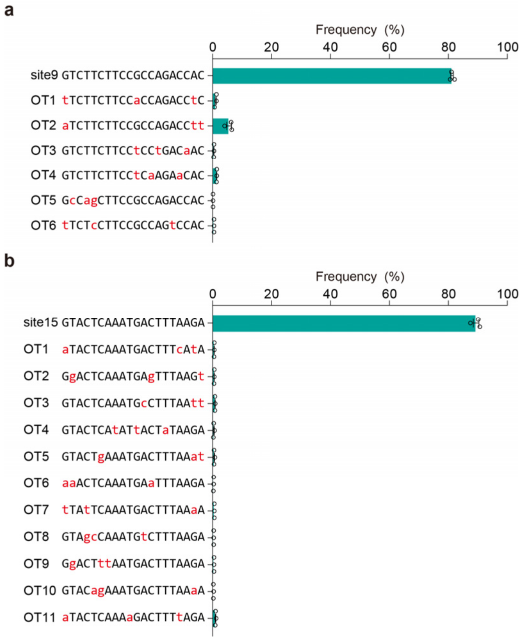

Supplementary Figure 7. The sgRNA-dependent off-target analysis for gTBEv3 at more sites.

The sgRNA-dependent off-target analysis for gTBEv3 editing at site 9 (**a**) and site 15 (**b**) (*n* = 3 independent biological replicates). OT: off-target. All values are presented as mean ± s.e.m. Source data are provided as a Source Data file.

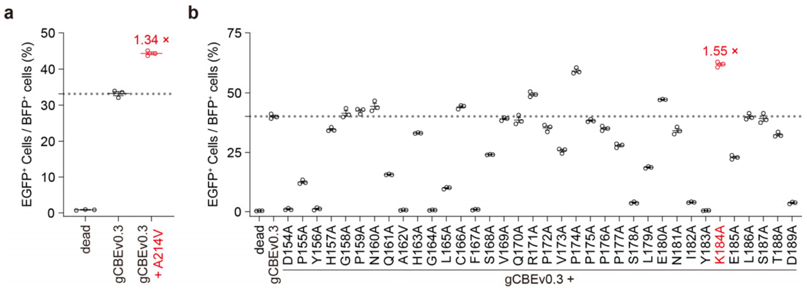

Supplementary Figure 8. Performance of engineered variants in the gCBEv0.3 background.

a, Percentage of EGFP$^+$ cells of gCBE variant by introduction of the mutation A214V (n = 3 independent biological replicates). **b**, Percentage of EGFP$^+$ cells of gCBE variants from alanine-scanning mutagenesis of regions covering the catalytic water-activating loop and the Pro-rich loop (n = 3 independent biological replicates). Replacement of alanine with valine (A>V) is intended to cover all the residues in the interested regions. dead, catalytically inactive UNG2Δ88 (carrying D154N and H277N mutations, equivalent to D145N and H268N of UNG1). All values are presented as mean ± s.e.m. Source data are provided as a Source Data file.

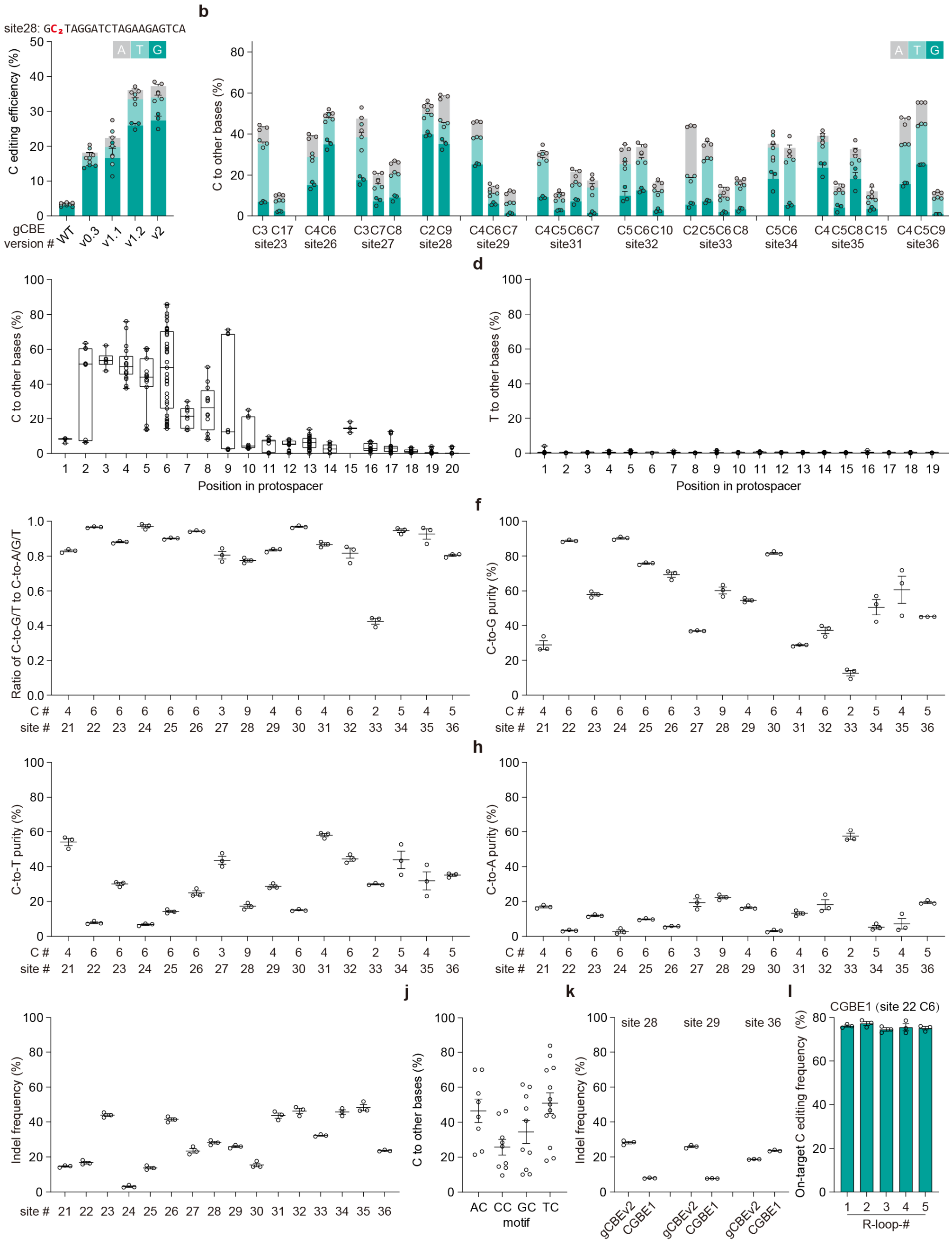

Supplementary Figure 9. Further characterization of editing profiles for gCBEv2.
a, Frequency of C base editing outcomes with different gCBE variants at the edited C2 position in site 28 in transfected HEK293T cells by target deep sequencing (mean ± s.e.m., $n = 3$ independent biological replicates). **b**, Stacked bar plots showing the on-target DNA base editing at two or more positions with C conversion frequencies >10% at each genomic site in HEK293T cells (mean ± s.e.m., $n = 3$ independent biological replicates). **c,d**, Frequencies of C (**c**) and T (**d**)

conversions by gCBEv2 across the protospacer positions 1-20 (where PAM is at positions 21–23) from the edited sites in Figure 4c. Single dot represents individual replicate (*n* = 3 independent replicates per site), and boxes span the interquartile range (25th to 75th percentile); horizontal lines within the boxes indicate the median (50%); and whiskers extend to the minimal and maximal values. **e**, The ratio of C-to-G/T to C-to-A/G/T conversion frequency by gCBEv2 editing at the sites shown in Figure 4c. **f-h**, Percentage of C-to-G (**f**), C-to-T (**g**) or C-to-A (**h**) editing by gCBEv2 at various edited sites shown in Figure 3a (mean ± s.e.m., *n* = 3 independent biological replicates). **i**, indels frequencies with gCBEv2 at 16 on-target sites (mean ± s.e.m., *n* = 3 independent biological replicates). In **e-i**, C#: C position with highest on-target base editing frequencies across protospacer positions 1-20. site #: genomic site number. **j**, The statistical analysis of on-target DNA base editing for each NC motif from the 16 edited sites. Each dot represents the mean of three biological replicates for each edited position at various edited sites. *n* = 8, 9, 10, 13 for motif AC, CC, GC, TC. **k**, indels frequencies with gCBEv2 and CGBE1 at 3 on-target sites from Figure 4d (mean ± s.e.m., *n* = 3 independent biological replicates). **l**, On-target base editing frequencies for CGBE1 at C6 of site 22 in HEK293T cells for the orthogonal R-loop assay (mean ± s.e.m., *n* = 3 independent biological replicates). Source data are provided as a Source Data file.

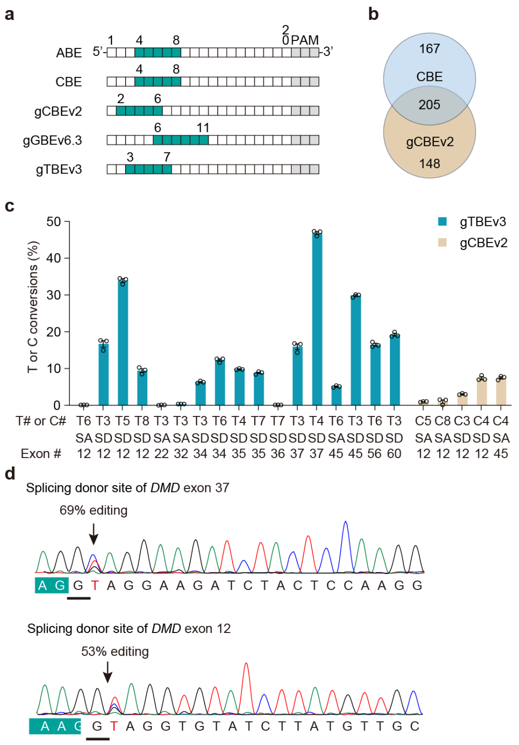

Supplementary Figure 10. Base editing at spicing sites with gTBEv3.

a, The optimal editing windows for various base editors. PAM, Protospacer adjacent motif. **b**, Venn diagram showing the distribution of sgRNAs for CBE and gCBEv2 in Figure 5b. **c**, Bar plots showing the frequency of T or C conversations at several splicing acceptor (SA) or splicing donor (SD) sites of interest targeted by gTBEv3 or gCBEv2 (mean ± s.e.m., n = 3 independent biological replicates). T# or C#: The position of targeted T or C across protospacer positions 1–20. **d**, DNA sequencing chromatograms for targeting the SD site of human *DMD* exon 37 and exon 12 with gTBEv3. Sanger sequencing results were quantified by EditR. Source data are provided as a Source Data file.

a

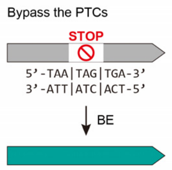

b

From:	TAA	TAG	TGA
ABE	TGG, CAA	TGG, CAG	TGG, CGA
CBE	-	-	-
CGBE	-	TAC	TCA
AYBE	TCA, TTA, TAC, TAT, AAA, GAA	TCG, TTG, AAG, GAG	TGC, TGT, AGA, GGA
gCBE	-	TAC	TCA
gGBE	-	TAC, TAT	TCA, TTA
gTBE	CAA, GAA, TCA, TAC, TGG, TCG	CAG, GAG, TCG, TGG	CGA, GGA, TGC, TGG

c

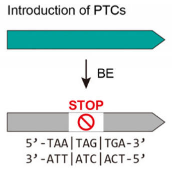

d

To:	TAA	TAG	TGA
ABE	-	-	-
CBE	CAA, TGG	CAG, TGG	CGA, TGG
CGBE	-	TAC	TCA
AYBE	AAA, TAT,	AAG, TTG	AGA, TGT
gCBE	-	TAC	TCA
gGBE	GAA, TAC, TCA	GAG, TCG	GGA, TGC
gTBE	-	TAT	TTA

e

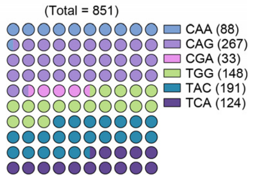

Supplementary Figure 11. PTCs editing and introduction for various base editors.
a, principle for bypassing premature termination codons (PTCs) with various base editors. **b**, the possible codon outcomes from stop codons (TAA, TAG or TGA) editing with different base editors. **c**, principle for introduction of PTCs with various base editors. **d**, the available codons for editing into stop codons (TAA, TAG or TGA) with different base editors. **e**, The 10 × 10 dot plot diagram showing the percentage of possible sgRNAs for introduction of premature termination codons (PTCs) by targeting different codons (with the number of available sgRNAs presented in the right) in 15 well-studied genes (*AGT, ANGPTL3, APOC3, B2M, CD33, DNMT3A, HPD, KLKB1, PCSK9, PDCD1, PRDM1, TGFBR2, TRAC, TTR, VEGFA*) for gene and cell therapy research with gGBEv6.3 and CBE. In **b** and **d**, AYBE, AYBEv3; gCBE, gCBEv2; gGBE, gGBEv6.3; gTBE, gTBEv3. Source data are provided as a Source Data file.

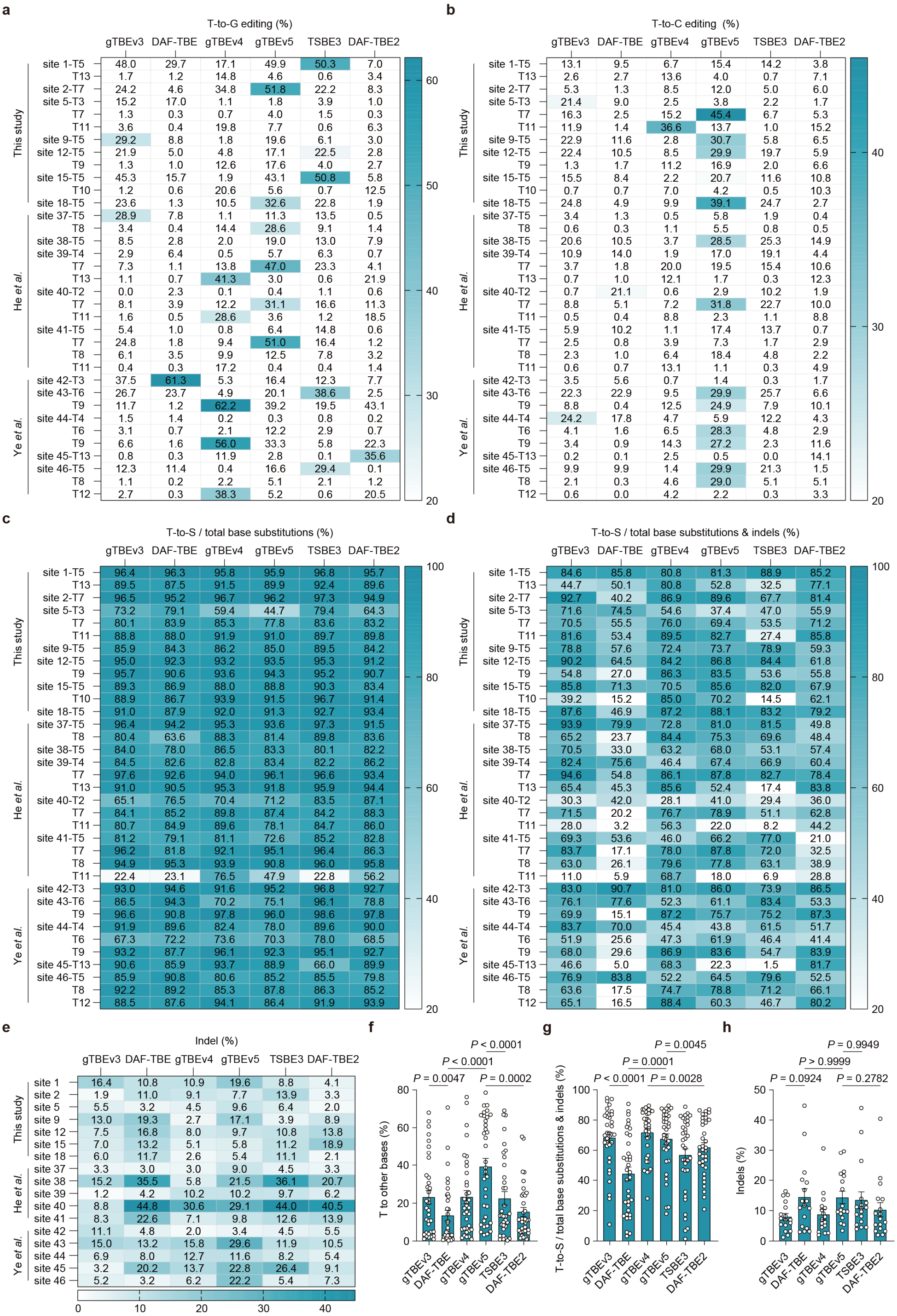
a
T-to-G editing (%)
gTBEv3 DAF-TBE gTBEv4 gTBEv5 TSBE3 DAF-TBE2
This study
He et al.
Ye et al.
site 1-T5 48.0 29.7 17.1 49.9 50.3 7.0
T13 1.7 1.2 14.8 4.6 0.6 3.4
site 2-T7 24.2 4.6 34.8 51.8 22.2 8.3
site 5-T3 15.2 17.0 1.1 1.8 3.9 1.0
T7 1.3 0.3 0.7 4.0 1.5 0.3
T11 3.6 0.4 19.8 7.7 0.6 6.3
site 9-T5 29.2 8.8 1.8 19.6 6.1 3.0
site 12-T5 21.9 5.0 4.8 17.1 22.5 2.8
T9 1.3 1.0 12.6 17.6 4.0 2.7
site 15-T5 45.3 15.7 1.9 43.1 50.8 5.8
T10 1.2 0.6 20.6 5.6 0.7 12.5
site 18-T5 23.6 1.3 10.5 32.6 22.8 1.9
site 37-T5 28.9 7.8 1.1 11.3 13.5 0.5
T8 3.4 0.4 14.4 28.6 9.1 1.4
site 38-T5 8.5 2.8 2.0 19.0 13.0 7.9
site 39-T4 2.9 6.4 0.5 5.7 6.3 0.7
T7 7.3 1.1 13.8 47.0 23.3 4.1
T13 1.1 0.7 41.3 3.0 0.6 21.9
site 40-T2 0.0 2.3 0.1 0.4 1.1 0.6
T7 8.1 3.9 12.2 31.1 16.6 11.3
T11 1.6 0.5 28.6 3.6 1.2 18.5
site 41-T5 5.4 1.0 0.8 6.4 14.8 0.6
T7 24.8 1.8 9.4 51.0 16.4 1.2
T8 6.1 3.5 9.9 12.5 7.8 3.2
T11 0.4 0.3 17.2 0.4 0.4 1.4
site 42-T3 37.5 61.3 5.3 16.4 12.3 7.7
site 43-T6 26.7 23.7 4.9 20.1 38.6 2.5
T9 11.7 1.2 62.2 39.2 19.5 43.1
site 44-T4 1.5 1.4 0.2 0.3 0.8 0.2
T6 3.1 0.7 2.1 12.2 2.9 0.7
T9 6.6 1.6 56.0 33.3 5.8 22.3
site 45-T13 0.8 0.3 11.9 2.8 0.1 35.6
site 46-T5 12.3 11.4 0.4 16.6 29.4 0.1
T8 1.1 0.2 2.2 5.1 2.1 1.2
T12 2.7 0.3 38.3 5.2 0.6 20.5
60 50 40 30 20
b
T-to-C editing (%)
gTBEv3 DAF-TBE gTBEv4 gTBEv5 TSBE3 DAF-TBE2
This study
He et al.
Ye et al.
site 1-T5 13.1 9.5 6.7 15.4 14.2 3.8
T13 2.6 2.7 13.6 4.0 0.7 7.1
site 2-T7 5.3 1.3 8.5 12.0 5.0 6.0
site 5-T3 21.4 9.0 2.5 3.8 2.2 1.7
T7 16.3 2.5 15.2 45.4 6.7 5.3
T11 11.9 1.4 36.6 13.7 1.0 15.2
site 9-T5 22.9 11.6 2.8 30.7 5.8 6.5
site 12-T5 22.4 10.5 8.5 29.9 19.7 5.9
T9 1.3 1.7 11.2 16.9 2.0 6.6
site 15-T5 15.5 8.4 2.2 20.7 11.6 10.8
T10 0.7 0.7 7.0 4.2 0.5 10.3
site 18-T5 24.8 4.9 9.9 39.1 24.7 2.7
site 37-T5 3.4 1.3 0.5 5.8 1.9 0.4
T8 0.6 0.3 1.1 5.5 0.8 0.5
site 38-T5 20.6 10.5 3.7 28.5 25.3 14.9
site 39-T4 10.9 14.0 1.9 17.0 19.1 4.4
T7 3.7 1.8 20.0 19.5 15.4 10.6
T13 0.7 1.0 12.1 1.7 0.3 12.3
site 40-T2 0.7 21.1 0.6 2.9 10.2 1.9
T7 8.8 5.1 7.2 31.8 22.7 10.0
T11 0.5 0.4 8.8 2.3 1.1 8.8
site 41-T5 5.9 10.2 1.1 17.4 13.7 0.7
T7 2.5 0.8 3.9 7.3 1.7 2.9
T8 2.3 1.0 6.4 18.4 4.8 2.2
T11 0.6 0.7 13.1 1.1 0.3 4.9
site 42-T3 3.5 5.6 0.7 1.4 0.3 1.7
site 43-T6 22.3 22.9 9.5 29.9 25.7 6.6
T9 8.8 0.4 12.5 24.9 7.9 10.1
site 44-T4 24.2 17.8 4.7 5.9 12.2 4.3
T6 4.1 1.6 6.5 28.3 4.8 2.9
T9 3.4 0.9 14.3 27.2 2.3 11.6
site 45-T13 0.2 0.1 2.5 0.5 0.0 14.1
site 46-T5 9.9 9.9 1.4 29.9 21.3 1.5
T8 2.1 0.3 4.6 29.0 5.1 5.1
T12 0.6 0.0 4.2 2.2 0.3 3.3
40 30 20
c
T-to-S / total base substitutions (%)
gTBEv3 DAF-TBE gTBEv4 gTBEv5 TSBE3 DAF-TBE2
This study
He et al.
Ye et al.
site 1-T5 96.4 96.3 95.8 95.9 96.8 95.7
T13 89.5 87.5 91.5 89.9 92.4 89.6
site 2-T7 96.5 95.2 96.7 96.2 97.3 94.9
site 5-T3 73.2 79.1 59.4 44.7 79.4 64.3
T7 80.1 83.9 85.3 77.8 83.6 83.2
T11 88.8 88.0 91.9 91.0 89.7 89.8
site 9-T5 85.9 84.3 86.2 85.0 89.5 84.2
site 12-T5 95.0 92.3 93.2 93.5 95.3 91.2
T9 95.7 90.6 93.6 94.3 95.2 90.7
site 15-T5 89.3 86.9 88.0 88.8 90.3 83.4
T10 88.9 86.7 93.9 91.5 96.7 91.4
site 18-T5 91.0 87.9 92.0 91.3 92.7 93.4
site 37-T5 96.4 94.2 95.3 93.6 97.3 91.5
T8 80.4 63.6 88.3 81.4 89.8 83.6
site 38-T5 84.0 78.0 86.5 83.3 80.1 82.2
site 39-T4 84.5 82.6 82.8 83.4 82.2 86.2
T7 97.6 92.6 94.0 96.1 96.6 93.4
T13 91.0 90.5 95.3 91.8 95.9 94.4
site 40-T2 65.1 76.5 70.4 71.2 83.5 87.1
T7 84.1 85.2 89.8 87.4 84.2 88.3
T11 80.7 84.9 89.6 78.1 84.7 86.0
site 41-T5 81.2 79.1 81.1 72.6 85.2 82.8
T7 96.2 81.8 92.1 95.1 96.4 86.3
T8 94.9 95.3 93.9 90.8 96.0 95.8
T11 22.4 23.1 76.5 47.9 22.8 56.2
site 42-T3 93.0 94.6 91.6 95.2 96.8 92.7
site 43-T6 86.5 94.3 70.2 75.1 96.1 78.8
T9 96.6 90.8 97.8 96.0 98.6 97.8
site 44-T4 91.9 89.6 82.4 78.0 89.6 90.0
T6 67.3 72.2 73.6 70.3 78.0 68.5
T9 93.2 87.7 96.1 92.3 95.1 92.7
site 45-T13 90.6 85.9 93.7 88.9 66.0 89.9
site 46-T5 85.9 90.8 80.6 85.2 85.5 79.8
T8 92.2 89.2 85.3 87.8 86.3 85.2
T12 88.5 87.6 94.1 86.4 91.9 93.9
100 80 60 40 20
d
T-to-S / total base substitutions & indels (%)
gTBEv3 DAF-TBE gTBEv4 gTBEv5 TSBE3 DAF-TBE2
This study
He et al.
Ye et al.
site 1-T5 84.6 85.8 80.8 81.3 88.9 85.2
T13 44.7 50.1 80.8 52.8 32.5 77.1
site 2-T7 92.7 40.2 86.9 89.6 67.7 81.4
site 5-T3 71.6 74.5 54.6 37.4 47.0 55.9
T7 70.5 55.5 76.0 69.4 53.5 71.2
T11 81.6 53.4 89.5 82.7 27.4 85.8
site 9-T5 78.8 57.6 72.4 73.7 78.9 59.3
site 12-T5 90.2 64.5 84.2 86.8 84.4 61.8
T9 54.8 27.0 86.3 83.5 53.6 55.8
site 15-T5 85.8 71.3 70.5 85.6 82.0 67.9
T10 39.2 15.2 85.0 70.2 14.5 62.1
site 18-T5 87.6 46.9 87.2 88.1 83.2 79.2
site 37-T5 93.9 79.9 72.8 81.0 81.5 49.8
T8 65.2 23.7 84.4 75.3 69.6 48.4
site 38-T5 70.5 33.0 63.2 68.0 53.1 57.4
site 39-T4 82.4 75.6 46.4 67.4 66.9 60.4
T7 94.6 54.8 86.1 87.8 82.7 78.4
T13 65.4 45.3 85.6 52.4 17.4 83.8
site 40-T2 30.3 42.0 28.1 41.0 29.4 36.0
T7 71.5 20.2 76.7 78.9 51.1 62.8
T11 28.0 3.2 56.3 22.0 8.2 44.2
site 41-T5 69.3 53.6 46.0 66.2 77.0 21.0
T7 83.7 17.1 78.0 87.8 72.0 32.5
T8 63.0 26.1 79.6 77.8 63.1 38.9
T11 11.0 5.9 68.7 18.0 6.9 28.8
site 42-T3 83.0 90.7 81.0 86.0 73.9 86.5
site 43-T6 76.1 77.6 52.3 61.1 83.4 53.3
T9 69.9 15.1 87.2 75.7 75.2 87.3
site 44-T4 83.7 70.0 45.4 43.8 61.5 51.7
T6 51.9 25.6 47.3 61.9 46.4 41.4
T9 68.0 29.6 86.9 83.6 54.7 83.9
site 45-T13 46.6 5.0 68.3 22.3 1.5 81.7
site 46-T5 76.9 83.8 52.2 64.5 79.6 52.5
T8 63.6 17.5 74.7 78.8 71.2 66.1
T12 65.1 16.5 88.4 60.3 46.7 80.2
100 80 60 40 20
e
Indel (%)
gTBEv3 DAF-TBE gTBEv4 gTBEv5 TSBE3 DAF-TBE2
This study
He et al.
Ye et al.
site 1 16.4 10.8 10.9 19.6 8.8 4.1
site 2 1.9 11.0 9.1 7.7 13.9 3.3
site 5 5.5 3.2 4.5 9.6 6.4 2.0
site 9 13.0 19.3 2.7 17.1 3.9 8.9
site 12 7.5 16.8 8.0 9.7 10.8 13.8
site 15 7.0 13.2 5.1 5.8 11.2 18.9
site 18 6.0 11.7 2.6 5.4 11.1 2.1
site 37 3.3 3.0 3.0 9.0 4.5 3.3
site 38 15.2 35.5 5.8 21.5 36.1 20.7
site 39 1.2 4.2 10.2 10.2 9.7 6.2
site 40 8.8 44.8 30.6 29.1 44.0 40.5
site 41 8.3 22.6 7.1 9.8 12.6 13.9
site 42 11.1 4.8 2.0 3.4 4.5 5.5
site 43 15.0 13.2 15.8 29.6 11.9 10.5
site 44 6.9 8.0 12.7 11.6 8.2 5.4
site 45 3.2 20.2 13.7 22.8 26.4 9.1
site 46 5.2 3.2 6.2 22.2 5.4 7.3
0 10 20 30 40
f
P < 0.0001
P < 0.0001
P = 0.0047
P = 0.0002
T to other bases (%)
80 60 40 20 0
gTBEv3 DAF-TBE gTBEv4 gTBEv5 TSBE3 DAF-TBE2
g
P = 0.0045
P = 0.0001
P < 0.0001
P = 0.0028
T-to-S / total base substitutions & indels (%)
100 80 60 40 20 0
gTBEv3 DAF-TBE gTBEv4 gTBEv5 TSBE3 DAF-TBE2
h
P = 0.9949
P > 0.9999
P = 0.0924
P = 0.2782
Indels (%)
50 40 30 20 10 0
gTBEv3 DAF-TBE gTBEv4 gTBEv5 TSBE3 DAF-TBE2

Supplementary Figure 12. Additional comparison of different gTBEs.

a,b, The frequencies of T-to-G (**a**) or T-to-G (**b**) conversions. The highest frequencies (>20%) of edited thymines at corresponding positions were highlighted as Heat map. **c,d**, Heat map showing the percentages of T-to-S to total base conversions (**c**) or T-to-S to total T editing (base conversions and indels; **d**) for various base editors. **e**, Heat map showing the indels frequencies for various base editors. For **a-e**, $n = 3$ independent biological replicates per site, note $n = 2$ for site 44 targeted by gTBEv4. **f-h**, The statistical analysis of T base editing (**f**, $n = 35$ positions), T-to-S percentages (**g**, $n = 35$ positions) and indels (**h**, $n = 17$ sites). All values are presented as mean ± s.e.m. Each dot represents the mean of three biological replicates for each edited position at various edited sites. **a-h**, the graphs were derived from the data for various base editors shown in Figure 6**c**. Dunnett's multiple comparisons test after one-way ANOVA was used to compare the gTBEv3 or gTBEv5 with other base editors in f–h. Source data are provided as a Source Data file.

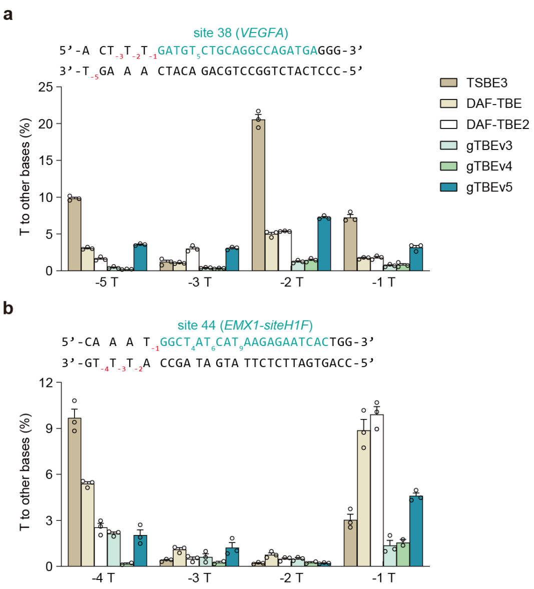

Supplementary Figure 13. T editing in the dsDNA upstream from the target site.

a, Bar plots showing the frequency of T conversation at -5T, -3T, -2T and -1T in the dsDNA upstream from the target site 38 (*VEGFA*) (n = 3 independent biological replicates). **b**, Bar plots showing the frequency of T conversation at -4T, -3T, -2T and -1T in the dsDNA upstream from the target site 44 (*EMX1-siteH1F*) (n = 3 independent biological replicates, note n = 2 for gTBEv4). All values are presented as mean ± s.e.m., the graphs were derived from the data for various base editors shown in Figure 6c. Source data are provided as a Source Data file.

a

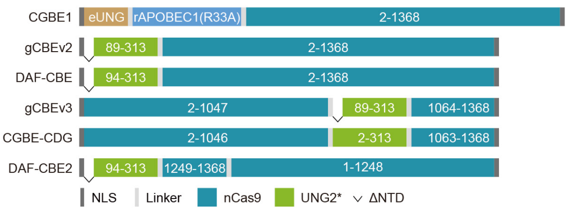

b C to other bases & indels (%)

Group	Site	CGBE1	gCBEv2	DAF-CBE	gCBEv3	CGBE-CDG	DAF-CBE2
This study	site 25-C6	72.0	61.3	7.7	80.9	43.2	7.7
	C9	20.8	9.0	1.5	54.2	7.5	22.2
	C12	8.5	11.5	3.2	32.1	7.8	56.8
	site 27-C3	2.9	49.4	71.0	49.5	51.9	5.1
	C7	49.0	24.6	6.0	48.2	16.7	2.8
	C8	13.2	27.8	8.3	60.1	24.6	11.9
	C12	11.7	8.8	6.4	24.1	7.7	49.5
	site 28-C2	3.2	59.5	61.0	56.8	62.2	33.0
	C9	44.1	68.4	43.0	75.5	31.3	56.8
	site 29-C4	3.3	63.0	54.6	23.1	25.4	5.2
	C6	83.3	32.3	18.0	36.1	15.7	5.7
	C7	4.1	29.7	18.9	39.9	18.7	9.7
	C8	4.7	25.6	19.5	41.6	15.8	18.0
	C10	10.4	19.9	19.6	40.8	12.0	24.3
	C13	5.0	17.9	17.9	23.3	10.0	51.0
	site 30-C6	67.2	76.5	31.0	59.4	36.7	17.7
	C13	1.1	12.4	7.0	18.0	10.7	65.5
	site 36-C4	79.0	36.9	46.5	29.2	39.2	6.3
	C5	25.7	47.0	52.3	63.8	38.0	14.5
	C9	5.7	13.2	8.8	42.1	11.4	12.1
	C10	6.8	8.7	7.3	29.3	8.9	11.2
	C11	6.0	12.1	10.3	23.2	10.4	38.5
	C12	5.0	10.1	9.5	17.4	9.9	38.6
	site 47-C5	13.2	78.1	70.8	80.6	58.3	16.2
	C12	10.0	9.4	5.0	17.7	4.0	50.4
	site 48-C5	2.0	32.2	28.0	37.8	16.9	5.2
	C10	8.9	13.9	23.9	36.7	15.0	26.2
	site 49-C4	1.3	68.7	67.3	65.9	41.6	3.5
	C10	33.8	15.6	9.2	64.3	6.8	15.2
	site 50-C4	0.4	22.1	15.9	33.3	17.7	2.1
	C5	1.7	45.5	21.3	54.0	18.5	4.3
	C13	0.6	7.4	16.4	11.2	3.3	39.2
He *et al.*	site 37-C3	1.0	36.6	30.5	30.6	16.3	2.9
	site 38-C6	85.5	73.4	57.4	78.8	56.7	23.5
	C9	4.4	42.8	12.2	78.7	26.0	44.5
	C13	2.6	10.4	7.3	18.1	8.4	59.4
	C14	2.2	9.3	5.5	17.2	6.2	50.8
	site 39-C6	72.6	67.4	21.4	77.8	46.6	9.7
	C11	3.7	15.4	12.6	34.6	8.1	67.1
	site 51-C6	48.0	64.0	35.2	74.4	42.1	9.4
	C11	3.6	22.7	13.2	50.9	14.5	56.5
	site 26-C4	27.1	72.5	78.6	73.8	68.3	17.4
	C6	75.5	78.5	31.0	85.8	58.9	41.2
	C11	8.1	27.8	23.8	42.0	17.3	87.7
Ye *et al.*	site 41-C3	6.1	62.3	70.3	34.1	67.8	10.7
	C6	87.8	54.1	10.4	79.7	20.4	5.8
	C12	3.4	14.9	9.7	22.8	11.5	64.6
	site 55-C3	1.8	69.4	73.8	48.2	56.5	17.2
	C7	1.1	41.5	5.4	47.9	3.6	4.3
	C12	1.1	18.7	12.0	24.4	6.0	64.2
	site 56-C4	3.1	51.3	64.7	47.4	53.5	5.6
	C6	70.4	38.1	9.0	70.9	25.3	4.7
	C12	1.9	12.8	5.7	20.0	8.8	50.7
	site 57-C3	6.4	32.4	44.5	15.5	20.0	4.1
	C6	2.2	59.0	25.1	64.8	16.0	6.4
	C12	1.6	17.3	10.8	23.4	6.2	60.5

Colour scale: 20, 40, 60, 80

c C to other bases (%)

Group	Site	CGBE1	gCBEv2	DAF-CBE	gCBEv3	CGBE-CDG	DAF-CBE2
This study	site 25-C6	68.2	54.1	7.0	51.3	38.7	4.8
	C9	14.8	1.7	0.7	25.9	2.7	15.9
	C12	1.2	3.8	2.2	5.8	2.8	43.4
	site 27-C3	2.5	43.2	68.9	27.8	45.7	4.3
	C7	47.1	18.2	3.0	24.5	9.0	1.3
	C8	10.1	21.0	4.7	36.0	16.2	9.8
	C12	7.6	3.4	1.9	4.3	0.9	45.5
	site 28-C2	2.1	48.9	53.4	40.8	52.6	28.9
	C9	42.3	51.6	28.8	48.8	18.3	48.2
	site 29-C4	2.1	44.8	40.4	8.2	14.6	2.5
	C6	81.0	13.9	2.4	19.2	4.8	2.2
	C13	1.4	4.6	2.7	8.4	2.7	41.5
	site 30-C6	66.1	69.1	28.8	43.9	22.9	16.4
	C13	0.2	6.5	4.9	5.8	0.5	63.1
	site 36-C4	76.0	32.3	43.2	20.0	33.8	4.0
	C5	22.0	41.4	48.1	52.7	31.5	10.9
	C9	0.9	6.4	3.5	28.2	3.8	6.1
	C11	0.9	3.5	3.8	5.8	2.1	25.1
	C12	0.5	3.9	4.4	3.4	3.1	30.3
	site 47-C5	12.0	75.6	68.4	75.9	55.3	13.3
	C12	8.3	6.8	2.0	14.4	1.1	44.2
	site 48-C5	1.6	21.5	9.6	25.1	5.0	2.0
	site 49-C4	0.9	59.8	60.4	57.3	36.2	2.6
	C10	32.6	10.9	5.0	55.8	2.4	12.4
	site 50-C5	1.6	28.0	6.3	33.4	9.1	2.5
	C13	0.1	0.8	0.4	1.4	0.2	30.5
He *et al.*	site 37-C3	0.9	35.2	28.4	26.5	14.9	2.5
	site 38-C6	83.5	68.8	55.0	68.3	51.8	15.4
	C9	2.0	36.8	8.9	65.5	20.3	31.2
	C13	0.3	4.7	3.8	6.1	3.4	35.0
	site 39-C6	71.2	65.0	20.4	72.9	43.7	8.3
	C11	0.8	11.6	10.7	29.4	5.1	63.5
	site 51-C6	46.9	60.7	33.3	63.8	38.9	8.2
	C11	2.2	19.2	11.2	36.9	11.0	54.0
	site 26-C4	21.5	52.6	62.2	53.3	53.7	11.2
	C6	69.8	61.1	15.0	65.3	44.8	34.1
	C11	1.5	11.3	9.0	23.0	5.1	77.1
Ye *et al.*	site 41-C3	4.7	53.4	66.5	19.0	60.9	9.0
	C6	84.9	42.4	4.7	59.7	11.5	2.4
	C12	0.6	3.6	2.8	6.5	2.5	58.8
	site 55-C3	1.7	63.5	71.6	30.4	54.9	16.0
	C7	0.9	33.3	2.5	29.7	2.2	2.5
	C12	0.1	10.9	4.1	14.6	4.7	59.9
	site 56-C4	2.5	45.4	61.1	32.2	47.4	5.1
	C6	69.2	30.8	4.8	53.9	18.1	3.9
	C12	0.2	5.0	2.7	7.2	2.5	48.7
	site 57-C3	5.8	22.7	37.9	8.4	16.9	3.1
	C6	1.5	51.8	17.7	59.1	13.0	5.1
	C12	0.1	9.5	2.7	16.8	3.0	57.3

Colour scale: 20, 40, 60, 80

d Indel (%)

Group	Site	CGBE1	gCBEv2	DAF-CBE	gCBEv3	CGBE-CDG	DAF-CBE2
This study	site 25	11.3	11.3	1.6	38.8	7.5	20.5
	site 27	7.2	11.5	10.3	31.3	12.5	6.8
	site 28	4.2	28.4	28.4	41.5	22.9	17.8
	site 29	9.1	24.8	29.9	28.4	13.5	15.7
	site 30	2.3	13.2	5.5	21.8	26.1	4.6
	site 36	12.3	19.4	19.5	31.9	26.0	20.9
	site 47	3.9	7.8	9.5	8.4	7.9	13.2
	site 48	1.7	25.6	51.9	26.0	28.0	10.9
	site 49	2.8	15.1	14.7	16.8	11.3	7.1
	site 50	1.0	24.5	39.2	27.6	12.4	17.5
He *et al.*	site 37	0.4	4.6	7.4	6.0	4.0	1.3
	site 38	5.2	12.3	8.1	22.9	13.5	39.9
	site 39	4.9	6.9	3.4	7.2	5.5	5.9
	site 51	3.2	7.9	6.0	21.9	7.9	5.6
	site 26	13.6	34.0	28.3	27.4	20.7	14.8
Ye *et al.*	site 41	5.4	20.0	13.3	30.0	16.3	9.5
	site 55	1.6	16.1	14.4	25.0	2.7	7.6
	site 56	3.8	14.9	8.2	22.7	12.4	4.5
	site 57	3.4	21.6	20.6	13.6	8.2	5.0

Colour scale: 0, 20, 40

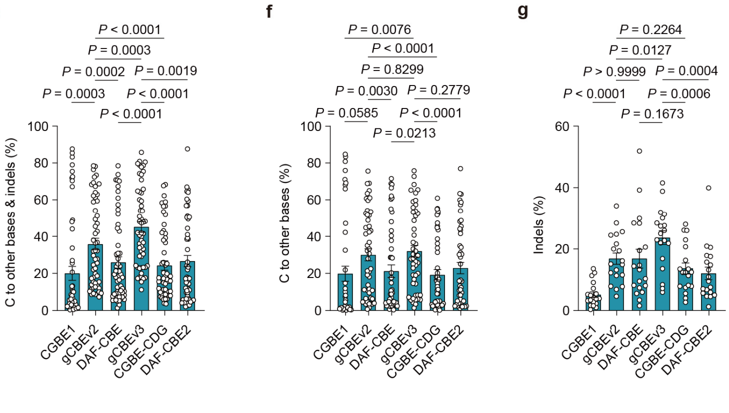

Supplementary Figure 14. Comparison of various glycosylase-based base editors for cytosine editing.

a, Schematic of the basic architectures for various base editors. The bipartite nuclear localization signal (bpNLS) is shown in dark gray, linker in light gray, and nCas9 in teal green. UNG2* (in light green), UNG2 variant from the corresponding base editor. ΔNTD, deletion of the N-terminal domain. **b**,**c**, The frequencies of total C editing (base conversions and indels, **b**) or C base conversions (**c**) for various base editors at 19 endogenous loci. The cytosines with editing frequencies >25% for any base editors were showed. The highest frequencies at corresponding positions were highlighted as Heat map (*n* = 3 independent biological replicates). **d**, Heat map showing the indels frequencies for various base editors (*n* = 3 independent biological replicates). **e-g**, The statistical analysis of total C editing (**e**, *n* = 56 positions), C base conversions (**f**, *n* = 49 positions) and indels (**g**, *n* = 19 sites). All values are presented as mean ± s.e.m. Each dot represents the mean of three biological replicates for each edited position at various edited sites. Dunnett's multiple comparisons test after one-way ANOVA was used to compare the gCBEv2 or gCBEv3 with other base editors. Source data are provided as a Source Data file.

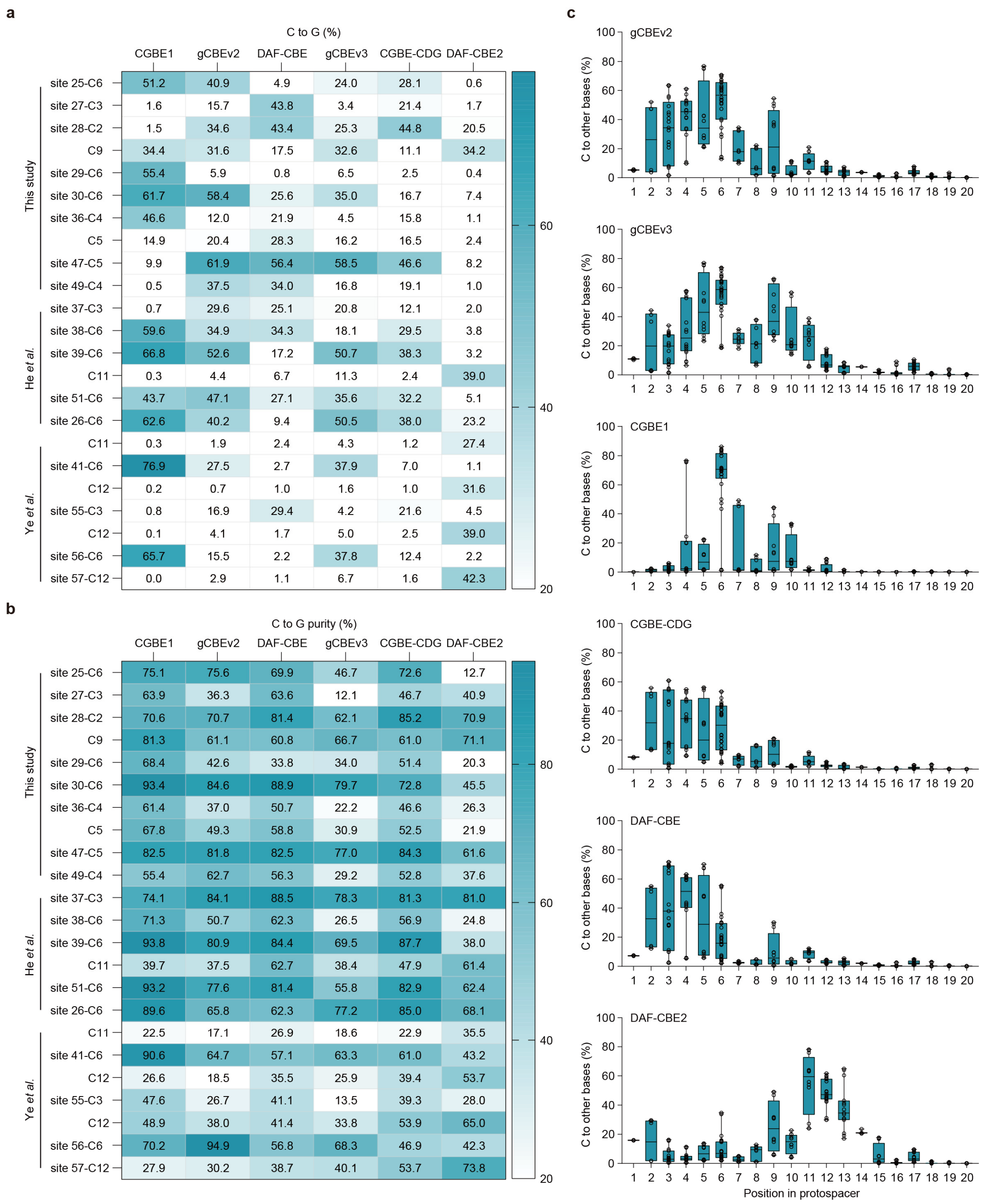

Supplementary Figure 15. Additional comparison of various glycosylase-based base editors for cytosine editing.

a,**b**, Heat map showing the C-to-G frequencies (**a**) or C-to-G purity (**b**) for various base editors (n = 3 independent biological replicates). The cytosines with C-to-G editing frequencies >25% for any base editors were highlighted. **c**, Frequencies of C base conversions by various base

editors across the protospacer positions 1-20 (where PAM is at positions 21–23). Single dot represents individual replicate (n = 3 independent replicates per site), and boxes span the interquartile range (25th to 75th percentile); horizontal lines within the boxes indicate the median (50%); and whiskers extend to the minimal and maximal values. The graphs were derived from the data for various base editors shown in Supplementary Figure 14**c**. Source data are provided as a Source Data file.

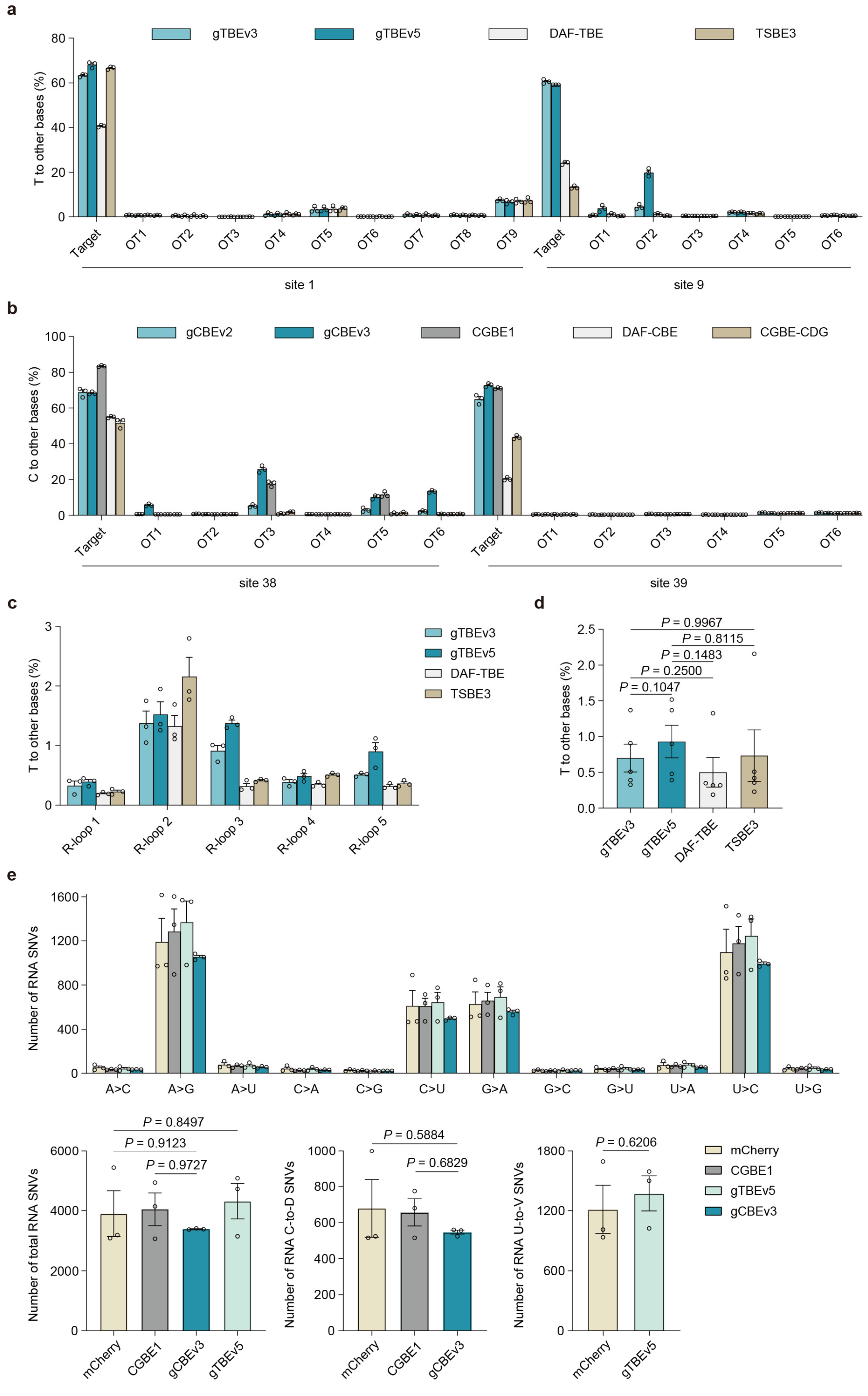

Supplementary Figure 16. Off-target analysis of various glycosylase-based base editors. **a**,**b**, The sgRNA-dependent off-target analysis of cumulative T editing (**a**) or C editing (**b**) frequencies for various base editors at corresponding sites (*n* = 3 independent biological replicates). OT: off-target. **c**, The sgRNA-independent cumulative off-target T editing detected

by the orthogonal R-loop assay at each R-loop site (*n* = 3 independent biological replicates). **d**, The statistical analysis of sgRNA-independent off-target T editing (*n* = 5 sites). Each dot represents the mean of three biological replicates for each edited position at various edited sites. Dunnett's multiple comparisons test after one-way ANOVA was used to compare the gTBEv3 or gTBEv5 with other base editors. **e**, RNA off-target analysis for various base editors (*n* = 3 independent biological replicates). The mCherry was used as control. D = A or G or U; V = A or C or G. For multiple comparisons, the Dunnett's multiple comparisons test after one-way ANOVA was used to compare the gCBEv3 or gTBEv5 with other groups. For comparison of RNA U-to-V SNVs for gTBEv5 and mCherry, two-tailed unpaired two-sample *t* test was used. All values are presented as mean ± s.e.m. Source data are provided as a Source Data file.

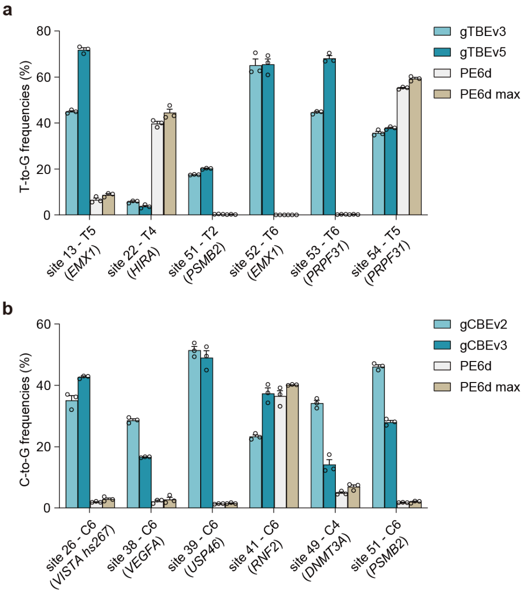

Supplementary Figure 17. Comparison between gTBEs or gCBEs and PEs.
a, Bar plots showing the on-target T-to-G editing frequency for various editors at each genomic site in HEK293T cells (*n* = 3 independent biological replicates). OT: off-target. **b**, Bar plots showing the on-target C-to-G editing frequency for various editors at each genomic site in HEK293T cells (*n* = 3 independent biological replicates). The PE6d was used together with epegRNA and nick sgRNA. For PE6d max, PE6d was co-expressed with the codon-optimized hMLH1dn, a dominant negative MMR protein. All values are presented as mean ± s.e.m. Source data are provided as a Source Data file.

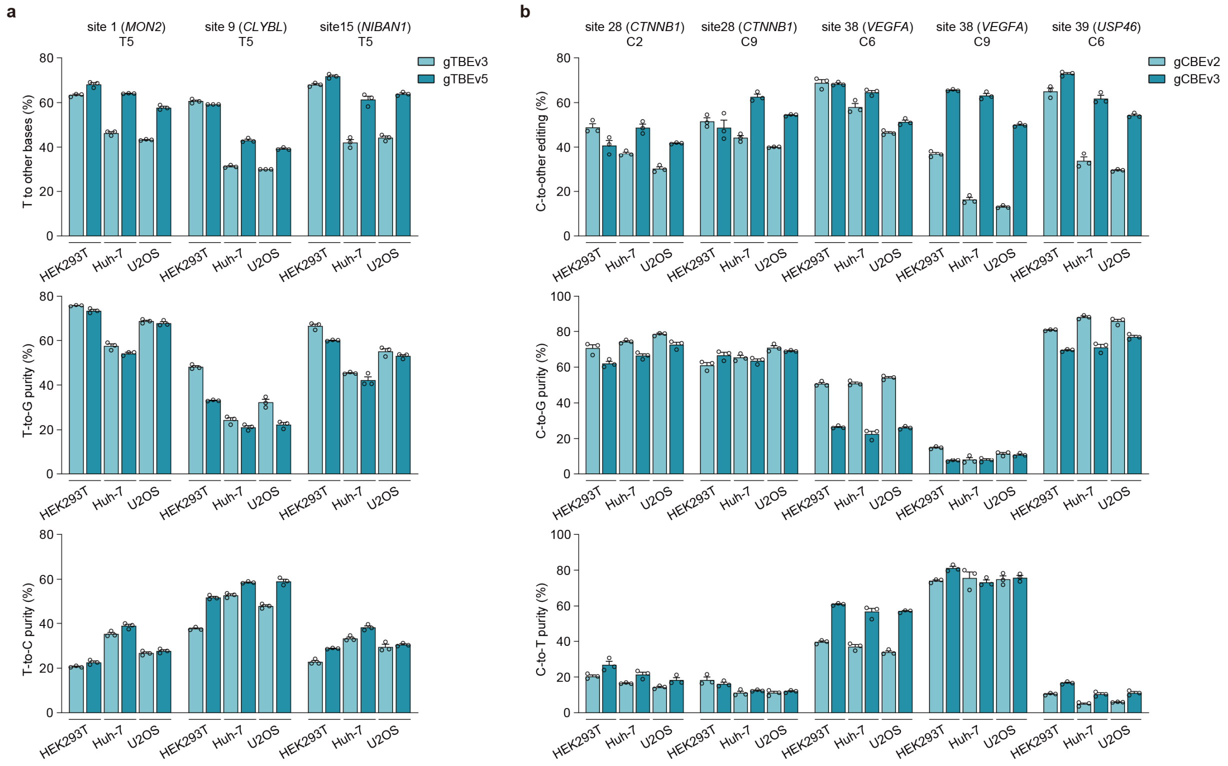

Supplementary Figure 18. Characterization of editing profiles for gTBEs or gCBEs in HEK293T, Huh-7, and U2OS cells.

a, Bar plots showing the on-target T base editing frequency, T-to-G editing purity or T-to-C editing purity for gTBEv3 and gTBEv5 in different cell lines (*n* = 3 independent biological replicates). **b**, Bar plots showing the on-target C base editing frequency, C-to-G editing purity or C-to-T editing purity for gCBEv2 and gCBEv3 in different cell lines (*n* = 3 independent biological replicates). Huh-7, a cell line established from a human hepatocellular carcinoma; U2OS, a cell line established from a human bone osteosarcoma. All values are presented as mean ± s.e.m. Source data are provided as a Source Data file.

Supplementary Tables

Supplementary Table 1. Protein sequence of gTBEv3 and gTBEv5 editor.

gTBEv3:

BpNLS-UNG2(Y156A,A214T,Q259A,Y284D)-Linker-nCas9(D10A)-BpNLS

MKRTADGSEFESPKKKRKVNVPVGFGESWKKHLSGEFGKPYFIKLMGFV
AEERKHYTVYPPPHQVFTWTQMCDIKDVKVVILGQDPAHGPNQAHGLC
FSVQRPVPPPPSLENIYKELSTDIEDFVHPGHGDLSGWAKQGVLLLNTVLT
VRAHQANSHKERGWEQFTDAVVSWLNQNSNGLVFLLWGSYAAKKGSA
IDRKRHHVLQTAHPSPLSVDRGFFGCRHFSKTNELLQKSGKKPIDWKELS
GGSSGGSSGSETPGTSESATPESSGGSSGGSDKKYSIGLAIGTNSVGWAVI
TDEYKVPSKKFKVLGNTDRHSIKKNLIGALLFDSGETAEATRLKRTARRR
YTRRKNRICYLQEIFSNEMAKVDDSFFHRLEESFLVEEDKKHERHPIFGNI
VDEVAYHEKYPTIYHLRKKLVDSTDKADLRLIYLALAHMIKFRGHFLIEG
DLNPDNSDVDKLFIQLVQTYNQLFEENPINASGVDAKAILSARLSKSRRLE
NLIAQLPGEKKNGLFGNLIALSLGLTPNFKSNFDLAEDAKLQLSKDTYDD
DLDNLLAQIGDQYADLFLAAKNLSDAILLSDILRVNTEITKAPLSASMIKR
YDEHHQDLTLLKALVRQQLPEKYKEIFFDQSKNGYAGYIDGGASQEEFY
KFIKPILEKMDGTEELLVKLNREDLLRKQRTFDNGSIPHQIHLGELHAILR
RQEDFYPFLKDNREKIEKILTFRIPYYVGPLARGNSRFAWMTRKSEETITP
WNFEEVVDKGASAQSFIERMTNFDKNLPNEKVLPKHSLLYEYFTVYNEL
TKVKYVTEGMRKPAFLSGEQKKAIVDLLFKTNRKVTVKQLKEDYFKKIE
CFDSVEISGVEDRFNASLGTYHDLLKIIKDKDFLDNEENEDILEDIVLTLTL
FEDREMIEERLKTYAHLFDDKVMKQLKRRRYTGWGRLSRKLINGIRDKQ
SGKTILDFLKSDGFANRNFMQLIHDDSLTFKEDIQKAQVSGQGDSLHEHI
ANLAGSPAIKKGILQTVKVVDELVKVMGRHKPENIVIEMARENQTTQKG
QKNSRERMKRIEEGIKELGSQILKEHPVENTQLQNEKLYLYYLQNGRDM
YVDQELDINRLSDYDVDHIVPQSFLKDDSIDNKVLTRSDKNRGKSDNVPS
EEVVKKMKNYWRQLLNAKLITQRKFDNLTKAERGGLSELDKAGFIKRQ
LVETRQITKHVAQILDSRMNTKYDENDKLIREVKVITLKSKLVSDFRKDF
QFYKVREINNYHHAHDAYLNAVVGTALIKKYPKLESEFVYGDYKVYDV
RKMIAKSEQEIGKATAKYFFYSNIMNFFKTEITLANGEIRKRPLIETNGETG
EIVWDKGRDFATVRKVLSMPQVNIVKKTEVQTGGFSKESILPKRNSDKLI
ARKKDWDPKKYGGFDSPTVAYSVLVVAKVEKGKSKKLKSVKELLGITI
MERSSFEKNPIDFLEAKGYKEVKKDLIIKLPKYSLFELENGRKRMLASAG
ELQKGNELALPSKYVNFLYLASHYEKLKGSPEDNEQKQLFVEQHKHYLD
EIIEQISEFSKRVILADANLDKVLSAYNKHRDKPIREQAENIIHLFTLTNLG
APAAFKYFDTTIDRKRYTSTKEVLDATLIHQSITGLYETRIDLSQLGGDSG
GSKRTADGSEFEPKKKRKV

gTBEv5:

BpNLS-Linker-nCas9(D10A, 2-1047)-Linker-UNG2(Y156A, A214T, Q259A, Y284D)-Linker-nCas9(D10A, 1064-1368)-BpNLS

MKRTADGSEFESPKKKRKVSGGSDKKYSIGLAIGTNSVGWAVITDEYKV
PSKKFKVLGNTDRHSIKKNLIGALLFDSGETAEATRLKRTARRRYTRRKN
RICYLQEIFSNEMAKVDDSFFHRLEESFLVEEDKKHERHPIFGNIVDEVAY
HEKYPTIYHLRKKLVDSTDKADLRLIYLALAHMIKFRGHFLIEGDLNPDN
SDVDKLFIQLVQTYNQLFEENPINASGVDAKAILSARLSKSRRLENLIAQL
PGEKKNGLFGNLIALSLGLTPNFKSNFDLAEDAKLQLSKDTYDDDLDNLL
AQIGDQYADLFLAAKNLSDAILLSDILRVNTEITKAPLSASMIKRYDEHHQ
DLTLLKALVRQQLPEKYKEIFFDQSKNGYAGYIDGGASQEEFYKFIKPILE
KMDGTEELLVKLNREDLLRKQRTFDNGSIPHQIHLGELHAILRRQEDFYP
FLKDNREKIEKILTFRIPYYVGPLARGNSRFAWMTRKSEETITPWNFEEVV
DKGASAQSFIERMTNFDKNLPNEKVLPKHSLLYEYFTVYNELTKVKYVT
EGMRKPAFLSGEQKKAIVDLLFKTNRKVTVKQLKEDYFKKIECFDSVEIS
GVEDRFNASLGTYHDLLKIIKDKDFLDNEENEDILEDIVLTLTLFEDREMI
EERLKTYAHLFDDKVMKQLKRRRYTGWGRLSRKLINGIRDKQSGKTILD
FLKSDGFANRNFMQLIHDDSLTFKEDIQKAQVSGQGDSLHEHIANLAGSP
AIKKGILQTVKVVDELVKVMGRHKPENIVIEMARENQTTQKGQKNSRER
MKRIEEGIKELGSQILKEHPVENTQLQNEKLYLYYLQNGRDMYVDQELDI
NRLSDYDVDHIVPQSFLKDDSIDNKVLTRSDKNRGKSDNVPSEEVVKKM
KNYWRQLLNAKLITQRKFDNLTKAERGGLSELDKAGFIKRQLVETRQIT
KHVAQILDSRMNTKYDENDKLIREVKVITLKSKLVSDFRKDFQFYKVREI
NNYHHAHDAYLNAVVGTALIKKYPKLESEFVYGDYKVYDVRKMIAKSE
QEIGKATAKYFFYSNIMNFFKSGGSSGGSSGSETPGTSESATPESSGGSSG
GSNVPVGFGESWKKHLSGEFGKPYFIKLMGFVAEERKHYTVYPPPHQVF
TWTQMCDIKDVKVVILGQDPAHGPNQAHGLCFSVQRPVPPPPSLENIYKE
LSTDIEDFVHPGHGDLSGWAKQGVLLLNTVLTVRAHQANSHKERGWEQ
FTDAVVSWLNQNSNGLVFLLWGSYAAKKGSAIDRKRHHVLQTAHPSPLS
VDRGFFGCRHFSKTNELLQKSGKKPIDWKELSGGSSGGSSGSETPGTSES
ATPESSGGSSGGSETNGETGEIVWDKGRDFATVRKVLSMPQVNIVKKTE
VQTGGFSKESILPKRNSDKLIARKKDWDPKKYGGFDSPTVAYSVLVVAK
VEKGKSKKLKSVKELLGITIMERSSFEKNPIDFLEAKGYKEVKKDLIIKLP
KYSLFELENGRKRMLASAGELQKGNELALPSKYVNFLYLASHYEKLKGS
PEDNEQKQLFVEQHKHYLDEIIEQISEFSKRVILADANLDKVLSAYNKHR
DKPIREQAENIIHLFTLTNLGAPAAFKYFDTTIDRKRYTSTKEVLDATLIHQ
SITGLYETRIDLSQLGGDSGGSKRTADGSEFEPKKKRKV
