# Supplementary material for: Development of deaminase-free T-to-S base editor and C-to-G base editor by engineered human uracil DNA glycosylase
Source: Nat Commun. 2024 Jun 8;15:4897. doi: 10.1038/s41467-024-49343-5 (PMC11162499; doi:10.1038/s41467-024-49343-5)
Supplement: Supplementary file 3 — Description of Additional Supplementary Files [file 41467_2024_49343_MOESM3_ESM.pdf]

Description of Additional Supplementary Files

File Name: Supplementary Data 1
Description: UNG variants in rounds of mutagenesis.

File Name: Supplementary Data 2
Description: sgRNA spacers, primers and target sequences used in this study.

File Name: Supplementary Data 3
Description: sgRNA candidates for editing the splicing sites with various base editors.

File Name: Supplementary Data 4
Description: sgRNA candidates for introduction of PTCs with various base editors.

File Name: Supplementary Data 5
Description: Differential gene expression (DEG) results of RNA-seq data.

File Name: Supplementary Data 6
Description: Comparison between gTBEs or gCBEs and PEs.
