# Supplementary material for: Development of deaminase-free T-to-S base editor and C-to-G base editor by engineered human uracil DNA glycosylase
Source: Nat Commun. 2024 Jun 8;15:4897. doi: 10.1038/s41467-024-49343-5 (PMC11162499; doi:10.1038/s41467-024-49343-5)
Supplement: Supplementary file 10 — Reporting Summary [file 41467_2024_49343_MOESM10_ESM.pdf]

nature portfolio

Corresponding author(s): Hui Yang

Last updated by author(s): May 2, 2024

Reporting Summary

Nature Portfolio wishes to improve the reproducibility of the work that we publish. This form provides structure for consistency and transparency in reporting. For further information on Nature Portfolio policies, see our Editorial Policies and the Editorial Policy Checklist.

Statistics

For all statistical analyses, confirm that the following items are present in the figure legend, table legend, main text, or Methods section.

n/a	Confirmed	
☐	☒	The exact sample size (*n*) for each experimental group/condition, given as a discrete number and unit of measurement
☐	☒	A statement on whether measurements were taken from distinct samples or whether the same sample was measured repeatedly
☐	☒	The statistical test(s) used AND whether they are one- or two-sided<br>*Only common tests should be described solely by name; describe more complex techniques in the Methods section.*
☒	☐	A description of all covariates tested
☒	☐	A description of any assumptions or corrections, such as tests of normality and adjustment for multiple comparisons
☐	☒	A full description of the statistical parameters including central tendency (e.g. means) or other basic estimates (e.g. regression coefficient) AND variation (e.g. standard deviation) or associated estimates of uncertainty (e.g. confidence intervals)
☐	☒	For null hypothesis testing, the test statistic (e.g. *F*, *t*, *r*) with confidence intervals, effect sizes, degrees of freedom and *P* value noted<br>*Give P values as exact values whenever suitable.*
☒	☐	For Bayesian analysis, information on the choice of priors and Markov chain Monte Carlo settings
☒	☐	For hierarchical and complex designs, identification of the appropriate level for tests and full reporting of outcomes
☒	☐	Estimates of effect sizes (e.g. Cohen's *d*, Pearson's *r*), indicating how they were calculated

Our web collection on statistics for biologists contains articles on many of the points above.

Software and code

Policy information about availability of computer code

Data collection	Next-generation sequencing data were obtained on the Illumina MiSeq platforms or Illumina NovaSeq 6000 platform.
Data analysis	FlowJo V10.5.3, Graphpad Prism(v8.3.0), Cutadapt(v0.6.6), FLASH (v1.2.11), fastx_toolkit (v0.0.14), CRISPResso2(v2.0.20b), Trimmomatic (v.0.39-2), Hisat2 (v.2.2.1), REDItools2, StringTie, DESeq2; Custom scripts for CRISPResso analyses are available on GitHub at https://github.com/yszhou2016/TBE.

For manuscripts utilizing custom algorithms or software that are central to the research but not yet described in published literature, software must be made available to editors and reviewers. We strongly encourage code deposition in a community repository (e.g. GitHub). See the Nature Portfolio guidelines for submitting code & software for further information.

Data

Policy information about availability of data

All manuscripts must include a data availability statement. This statement should provide the following information, where applicable:

- Accession codes, unique identifiers, or web links for publicly available datasets
- A description of any restrictions on data availability
- For clinical datasets or third party data, please ensure that the statement adheres to our policy

The high-throughput sequencing data generated in this study have been deposited in the National Center for Biotechnology Information Sequence Read Archive and can be accessed at https://www.ncbi.nlm.nih.gov/bioproject/PRJNA1105444. Source data are provided with this paper.

Research involving human participants, their data, or biological material

Policy information about studies with human participants or human data. See also policy information about sex, gender (identity/presentation), and sexual orientation and race, ethnicity and racism.

Reporting on sex and gender	*Use the terms sex (biological attribute) and gender (shaped by social and cultural circumstances) carefully in order to avoid confusing both terms. Indicate if findings apply to only one sex or gender; describe whether sex and gender were considered in study design; whether sex and/or gender was determined based on self-reporting or assigned and methods used.* *Provide in the source data disaggregated sex and gender data, where this information has been collected, and if consent has been obtained for sharing of individual-level data; provide overall numbers in this Reporting Summary. Please state if this information has not been collected.* *Report sex- and gender-based analyses where performed, justify reasons for lack of sex- and gender-based analysis.*
Reporting on race, ethnicity, or other socially relevant groupings	*Please specify the socially constructed or socially relevant categorization variable(s) used in your manuscript and explain why they were used. Please note that such variables should not be used as proxies for other socially constructed/relevant variables (for example, race or ethnicity should not be used as a proxy for socioeconomic status).* *Provide clear definitions of the relevant terms used, how they were provided (by the participants/respondents, the researchers, or third parties), and the method(s) used to classify people into the different categories (e.g. self-report, census or administrative data, social media data, etc.)* *Please provide details about how you controlled for confounding variables in your analyses.*
Population characteristics	*Describe the covariate-relevant population characteristics of the human research participants (e.g. age, genotypic information, past and current diagnosis and treatment categories). If you filled out the behavioural & social sciences study design questions and have nothing to add here, write "See above."*
Recruitment	*Describe how participants were recruited. Outline any potential self-selection bias or other biases that may be present and how these are likely to impact results.*
Ethics oversight	*Identify the organization(s) that approved the study protocol.*

Note that full information on the approval of the study protocol must also be provided in the manuscript.

Field-specific reporting

Please select the one below that is the best fit for your research. If you are not sure, read the appropriate sections before making your selection.

☒ Life sciences ☐ Behavioural & social sciences ☐ Ecological, evolutionary & environmental sciences

For a reference copy of the document with all sections, see nature.com/documents/nr-reporting-summary-flat.pdf

Life sciences study design

All studies must disclose on these points even when the disclosure is negative.

Sample size	No sample-size calculation was performed. Experiments were performed with three independent biological replicates unless otherwise noted, with the sample size numbers were listed in the corresponding figure legends. Sample sizes for these experiments were chosen based upon field standards and prior knowledge of experimental variation. (e.g. Gaudelli et al., Nature 2017; Koblan et al., Nat Biotechnol 2021)
Data exclusions	No data were excluded.
Replication	Data were obtained in the fashion of biological replications. We tested experimental conditions using different sgRNAs to ensure robustness. All the experimental results could be successfully reproduced. At least three independent biological replicates were performed.
Randomization	The experiments were conducted in a per cell-line basis. Therefore, the experiments were not randomized. For transfection, wells were randomly assigned.
Blinding	Blinding was not relevant to our study because it is not a subjective trial and NGS and other methods used for quantification in our study are not influenced by human interpretation and/or bias.

Reporting for specific materials, systems and methods

We require information from authors about some types of materials, experimental systems and methods used in many studies. Here, indicate whether each material, system or method listed is relevant to your study. If you are not sure if a list item applies to your research, read the appropriate section before selecting a response.

Materials & experimental systems

n/a	Involved in the study
☒	☐ Antibodies
☐	☒ Eukaryotic cell lines
☒	☐ Palaeontology and archaeology
☐	☒ Animals and other organisms
☒	☐ Clinical data
☒	☐ Dual use research of concern
☒	☐ Plants

Methods

n/a	Involved in the study
☒	☐ ChIP-seq
☐	☒ Flow cytometry
☒	☐ MRI-based neuroimaging

Eukaryotic cell lines

Policy information about cell lines and Sex and Gender in Research

Cell line source(s): HEK293T (Catalog# BNCC353535), Huh-7 (Catalog# BNCC337690) and U2OS (Catalog# BNCC352039) cell lines were purchased from BNCC.

Authentication: Cell lines were authenticated using STR profiling by the supplier.

Mycoplasma contamination: Cell lines have been tested negative for mycoplasma contamination.

Commonly misidentified lines (See ICLAC register): No commonly misidentified cell line listed in the database of ICLAC was used.

Animals and other research organisms

Policy information about studies involving animals; ARRIVE guidelines recommended for reporting animal research, and Sex and Gender in Research

Laboratory animals: humanized DMD females with human DMD exon 45 in C57BL/6 background (4 weeks old), C57BL/6 males (8 weeks old), ICR strain.

Wild animals: The study did not involve wild animals.

Reporting on sex: Super ovulated humanized DMD females with human DMD exon 45 in C57BL/6 background (4 weeks old) were mated with C57BL/6 males (8 weeks old), and females from the ICR strain were used as foster mothers.

Field-collected samples: The study did not involve samples collected from the field.

Ethics oversight: Experiments involving mice were approved by the Biomedical Research Ethics Committee of Center for HuidaGene Therapeutics Co. Ltd.

Note that full information on the approval of the study protocol must also be provided in the manuscript.

Plants

Seed stocks: *Report on the source of all seed stocks or other plant material used. If applicable, state the seed stock centre and catalogue number. If plant specimens were collected from the field, describe the collection location, date and sampling procedures.*

Novel plant genotypes: *Describe the methods by which all novel plant genotypes were produced. This includes those generated by transgenic approaches, gene editing, chemical/radiation-based mutagenesis and hybridization. For transgenic lines, describe the transformation method, the number of independent lines analyzed and the generation upon which experiments were performed. For gene-edited lines, describe the editor used, the endogenous sequence targeted for editing, the targeting guide RNA sequence (if applicable) and how the editor was applied.*

Authentication: *Describe any authentication procedures for each seed stock used or novel genotype generated. Describe any experiments used to assess the effect of a mutation and, where applicable, how potential secondary effects (e.g. second site T-DNA insertions, mosiacism, off-target gene editing) were examined.*

Flow Cytometry

Plots

Confirm that:

- ☒ The axis labels state the marker and fluorochrome used (e.g. CD4-FITC).
- ☒ The axis scales are clearly visible. Include numbers along axes only for bottom left plot of group (a 'group' is an analysis of identical markers).
- ☒ All plots are contour plots with outliers or pseudocolor plots.
- ☒ A numerical value for number of cells or percentage (with statistics) is provided.

Methodology

Sample preparation	To isolate cells, the transfected or non-transfected were dissociated enzymatically in an incubation solution of 100 µL Trypsin-EDTA (0.05%) at 37°C for 5 min. The digestion was stopped by adding 1 ml of DMEM medium with 10% Fetal Bovine Serum (FBS). The cell suspension was centrifuged for 3 min (1000 rpm), and the pellet was resuspended in DMEM medium with 10% FBS. Finally, the cell suspension was filtered through a 40-µm cell strainer, and mCherry+ or mCherry+/EGFP+ cells were isolated by FACS.
Instrument	BD FACS Aria III or Beckman CytoFLEX S.
Software	FlowJo V10.5.3
Cell population abundance	Samples were found to be >95% pure when assessed with a second round of flow cytometry analysis.
Gating strategy	Gating strategy: 1) in FSC-A/SSC-A or FSC-H/SSC-H gate for living cells, 2) using the non-transfected and negative cells to define the gates for BFP+, EGFP+ and/or mCherry+ cells, 3) apply this gate to all samples.

☒ Tick this box to confirm that a figure exemplifying the gating strategy is provided in the Supplementary Information.
