# Supplementary material for: Development of deaminase-free T-to-S base editor and C-to-G base editor by engineered human uracil DNA glycosylase
Source: Nat Commun. 2024 Jun 8;15:4897. doi: 10.1038/s41467-024-49343-5 (PMC11162499; doi:10.1038/s41467-024-49343-5)
Supplement: Supplementary file 2 — Peer Review File [file 41467_2024_49343_MOESM2_ESM.pdf]

nature portfolio

Peer Review File

Development of deaminase-free T-to-S base editor and C-to-G base editor by engineered human uracil DNA glycosylase

Open Access This file is licensed under a Creative Commons Attribution 4.0 International License, which permits use, sharing, adaptation, distribution and reproduction in any medium or format, as long as you give appropriate credit to the original author(s) and the source, provide a link to the Creative Commons license, and indicate if changes were made. In the cases where the authors are anonymous, such as is the case for the reports of anonymous peer reviewers, author attribution should be to 'Anonymous Referee' followed by a clear attribution to the source work. The images or other third party material in this file are included in the article's Creative Commons license, unless indicated otherwise in a credit line to the material. If material is not included in the article's Creative Commons license and your intended use is not permitted by statutory regulation or exceeds the permitted use, you will need to obtain permission directly from the copyright holder. To view a copy of this license, visit http://creativecommons.org/licenses/by/4.0/.

Editorial Note: This manuscript has been previously reviewed at another journal that is not operating a transparent peer review scheme. This document only contains reviewer comments and rebuttal letters for versions considered at *Nature Communications.*

Reviewer #1 (Remarks to the Author):

I do not have any more concerns. All of the comments of reviewer 2 have been well addressed. I would recommend the publication of this well-revised manuscript.

Reviewer #2 (Remarks to the Author):

Using the previously submitted and reviewed manuscript as a reference, the authors have adequately fulfilled most of the concerns I brought up as a reviewer. I agree with publication.
